# Syntactic structures in motion: investigating word order variations in verb-final (Korean) and verb-initial (Tongan) languages

**DOI:** 10.3389/fpsyg.2024.1360191

**Published:** 2024-04-24

**Authors:** Katsuo Tamaoka, Shaoyun Yu, Jingyi Zhang, Yuko Otsuka, Hyunjung Lim, Masatoshi Koizumi, Rinus G. Verdonschot

**Affiliations:** ^1^School of Foreign Language Studies, Shanghai University, Shanghai, China; ^2^Graduate School of Humanities, Nagoya University, Nagoya, Japan; ^3^Department of Chinese and Bilingual Studies, Hong Kong Polytechnic University, Hong Kong, China; ^4^Organization for Global Collaboration Center for Language and Cultural Studies, University of Miyazaki, Miyazaki, Japan; ^5^Faculty of Foreign Studies, Sophia University, Tokyo, Japan; ^6^Faculty of International Studies, Yamaguchi Prefectural University, Yamaguchi, Japan; ^7^Graduate School of Arts and Letters, Tohoku University, Sendai, Japan; ^8^Neurobiology of Language, Max Planck Institute for Psycholinguistics, Nijmegen, Netherlands

**Keywords:** topicalization, scrambling, word order, verb (head)-final language, verb (head)-initial language

## Abstract

This study explored sentence processing in two typologically distinct languages: Korean, a verb-final language, and Tongan, a verb-initial language. The first experiment revealed that in Korean, sentences arranged in the scrambled OSV (Object, Subject, Verb) order were processed more slowly than those in the canonical SOV order, highlighting a scrambling effect. It also found that sentences with subject topicalization in the SOV order were processed as swiftly as those in the canonical form, whereas sentences with object topicalization in the OSV order were processed with speeds and accuracy comparable to scrambled sentences. However, since topicalization and scrambling in Korean use the same OSV order, independently distinguishing the effects of topicalization is challenging. In contrast, Tongan allows for a clear separation of word orders for topicalization and scrambling, facilitating an independent evaluation of topicalization effects. The second experiment, employing a maze task, confirmed that Tongan’s canonical VSO order was processed more efficiently than the VOS scrambled order, thereby verifying a scrambling effect. The third experiment investigated the effects of both scrambling and topicalization in Tongan, finding that the canonical VSO order was processed most efficiently in terms of speed and accuracy, unlike the VOS scrambled and SVO topicalized orders. Notably, the OVS object-topicalized order was processed as efficiently as the VSO canonical order, while the SVO subject-topicalized order was slower than VSO but faster than VOS. By independently assessing the effects of topicalization apart from scrambling, this study demonstrates that both subject and object topicalization in Tongan facilitate sentence processing, contradicting the predictions based on movement-based anticipation.

## 1 Introduction

In languages spoken worldwide, sentence structures vary, with the verb sometimes positioned at the beginning or the end of a sentence. Irrespective of the verb’s position, the topic of discourse often precedes it, a phenomenon known as *topicalization*. This flexibility allows for the selection of various topics within a sentence. For example, in English, the object ‘my brother’ in the sentence ‘I am proud of my brother’ can be topicalized to form ‘My brother, I am proud of.’ Chomsky (1977) proposed that a topicalized phrase (topicP) is moved to a higher position within a sentence, typically at the specifier (Spec) of a complementizer phrase (CP), thus creating a more complex syntactic structure. Similarly, in Japanese, Shibatani (1990) noted that topicP syntactically belongs to a CP positioned higher than an Inflectional Phrase (IP). Moreover, Kuroda (1987) suggested that object topicalization in Japanese involves topicalization and scrambling movements, further complicating the sentence structure. We generally assumed that increased structural complexity leads to a heavier processing load (Holmes and O’Regan, 1981; Ford, 1983; King and Just, 1991; Just et al., 1996; Caplan et al., 1998; Bates et al., 1999; Gibson, 2000; Sekerina, 2003). Therefore, this study aims to investigate the processing of topicalized sentences in Korean, a verb-final language, and Tongan, a verb-initial language. We focus on multiple word orders created through syntactic movement, with the goal of understanding the processing dynamics of topicalized sentences in these languages.

### 1.1 Scrambling and topicalization in Korean

Korean, the native language of both South Korea and North Korea, is estimated to have more than 80 million people in the world who speak Korean as a first, second, or heritage language (Pae, 2024). Korean is a verb-final language with the canonical order of subject, object, and verb (SOV). The order of subject and object is relatively flexible. A simple transitive sentence in Korean can have one of two basic orders: either SOV or OSV. Noun phrases (NPs) are marked by one of three case markers (or particles): the subject or nominative marker-이/가, −*i/ka* (NP_NOM_), the object or accusative marker-을/를, *−eul/leul* (NP_ACC_), or the topic marker-은/는, *−eun/neun* (NP_TOP_). In this study, we represent sentences in Korean using standard romanization. These combinations enable the creation of four types of sentences (Lee and Ramsey, 2000; Pae, 2024). These, with either SOV or OSV, are illustrated in Sentences (1) to (4). All sentences fundamentally carry the same meaning. Sentence (1) is the canonical order, which employs the scrambled order OSV, as shown in Sentence (2). Topicalization in Korean is achieved by adding the marker -*eun/neun* to a noun phrase, as illustrated in Sentence (3) with subject topicalization and Sentence (4) with object topicalization.

(1) SOV: Canonical order.

*Eom-ma -ga sa-gwa -leul meog -eoss-da*.

NP(mother) NOM NP(apple) ACC V(eat) PST.

엄마가 사과를 먹었다.

‘(My) mother ate (an) apple.’

(2) OSV: Scrambled order.

*Sa-gwa -leul eom-ma -ga meog -eoss-da*.

NP(apple) ACC NP(mother) NOM V(eat) PST.

사과를 엄마가 먹었다.

(3) SOV: Subject topicalized order.

*Eom-ma -neun sa-gwa -leul meog -eoss-da*.

NP(mother) TOP NP(apple) ACC V(eat) PST.

엄마는 사과를 먹었다.

(4) OSV: Object topicalized order.

*Sa-gwa -neun eom-ma -ga meog -eoss-da*.

NP(apple) TOP NP(mother) NOM V(eat) PST.

사과는 엄마가 먹었다.

In Sentence (1), ‘(My) mother ate (an) apple,’ follows the SOV order with ‘(my) mother’ marked by the nominative case marker -*ga* (NP_NOM_), ‘(an) apple’ marked by the accusative case marker -*leul* (NP_ACC_), and the past tense verb (V-PST) *meog-eoss-da* ‘ate’ at the end. In Sentence (2), the positions of NP_NOM_ and NP_ACC_ are scrambled, as is characteristic of the OSV order. Once again, the final verb ‘ate’ appears at the end of the sentence. The OSV order is formed by moving the object to the beginning of the sentence. This word order was termed *scrambled* by Ross (1967), who primarily discussed this phenomenon in relation to Germanic languages (Neeleman, 1994; Broekhuis, 2008).

Several psycholinguistic studies conducted in Japanese, which shares similar syntactic features with Korean, have investigated sentence processing. These studies (e.g., Mazuka et al., 2002; Ueno and Kluender, 2003; Koizumi and Tamaoka, 2004, 2010; Miyamoto and Takahashi, 2004; Tamaoka et al., 2005, 2014; Imamura et al., 2016; Witzel and Witzel, 2016; Tamaoka and Mansbridge, 2019) consistently found that *the canonical SOV order is processed faster than the scrambled OSV order*. For instance, in the methodology employed by Tamaoka et al. (2005), participants were tasked with evaluating the correctness of each sentence, considering both its semantic coherence and grammatical accuracy. This approach is hereafter referred to as a ‘sentence correctness decision task.’ Utilizing this task, the study measured the processing time for both canonical SOV and scrambled OSV sentence orders in Japanese, employing sentences analogous to Korean Sentences (1) and (2). Without any preceding context, the study found that Japanese canonical SOV sentences were processed both more rapidly and accurately compared to their scrambled OSV counterparts. Drawing on these results, the current study predicts a similar processing advantage for canonical sentences in Korean over their scrambled alternatives.

The processing inefficiency observed in both accuracy and speed for scrambled sentences is commonly referred to as the *scrambling effect*. One potential explanation for the delay in processing the scrambled OSV order in Japanese comes from the *gap-filling parsing* model (Frazier and Rayner, 1982; Stowe, 1986; Frazier, 1987; Frazier and Clifton, 1989; Frazier and Flores D’Arcais, 1989). According to this model, native Korean speakers likely identify the initial NP_ACC_ marked by -leul as the filler and subsequently search for its original position in the specifier of the gap to establish the filler-gap dependency. Given that the OSV scrambled order entails a syntactically more complex structure than its corresponding SOV order, the process of gap-filling parsing for the OSV order is expected to be slower than for the SOV order. Therefore, we anticipate observing similar processing dynamics in Korean as those identified in Japanese, where scrambled sentences are processed less efficiently compared to canonical sentences.

In the processing of scrambled word order in Japanese and potentially in Korean, the filler-gap dependency is activated. While the subject typically appears first in a sentence, the object precedes it in a scrambled sentence. Given that Korean and Japanese are null-subject (or pro-drop) languages, the subject of a sentence can be omitted. Consequently, native speakers of Korean and Japanese may initially interpret the sentence as having a null subject. However, in a scrambled sentence, the subject follows the object (OS order). Native speakers of Korean and Japanese will then search for the *gap*_1_, representing the position where the object would occur in the SOV canonical order. They establish the filler-gap dependency between the object and the gap as O_1_ S *gap*_1_ before encountering the verb to fully comprehend the scrambled sentence. This additional processing step may prolong the processing time and potentially lead to comprehension errors.

In Korean, canonical and scrambled word orders overlap with topicalization orders. The subject in the SOV canonical order is typically positioned at the beginning of the sentence unless a pro-drop occurs. Therefore, the canonical S_NOM_OV and the topicalized S_TOP_OV share the same word order. For instance, S_NOM_OV for 경찰이 범인을 잡았다 (*Gyeong-chal-i beom-in-eul jab-ass-da*, ‘The police caught the culprit’) and S_TOP_OV for 경찰은 범인을 잡았다 (*Gyeong-chal-eun beom-in-eul jab-ass-da*) both have the subject in the initial position, which is canonical. It is important to note that Korean lacks definite and indefinite articles, so nouns in a sentence are typically denoted without an article. Similarly, O_ACC_SV and O_TOP_SV also share the same word order. For example, O_ACC_SV for 범인을 경찰이 잡았다 (*Beom-in-eul gyeong-chal-i jab-ass-da*, ‘The culprit, the police caught’) and O_TOP_SV for 범인은 경찰이 잡았다 (*Beom-in-eun gyeong-chal-i jab-ass-da*) both have the object in the scrambled position. Since the word orders of subject and object topicalization share the same pattern as canonical and scrambling word orders, it is difficult to measure the effect of subject and object topicalization independently from the processing of canonical and scrambling structures.

In Korean, noun phrases can be topicalized using the marker -*eun/neun* (NP_TOP_). As topicalization is a discourse feature, NP_TOP_ is positioned at the beginning of a sentence to indicate the topic. A subject or an object can be topicalized using the marker -*eun/neun*. Sentence (3) exemplifies a subject-topicalized (NP_SUB-TOP_) sentence in the SOV canonical order, beginning with ‘Speaking of (my) mother.’ Subject topicalization also implies an exclusionary meaning, referring specifically to ‘mother’ and not other family members. Sentence (4) illustrates an object-topicalized (NP_OBJ-TOP_) sentence in the OSV scrambled order, commencing with ‘Speaking of (the) apple.’ Object topicalization similarly implies an exclusionary meaning, specifically referring to ‘(the) apple’ and not other fruits.

It is also notable that the distinct grammatical particles (or makers) used for the subject (−이/가, *−i/ga*) and the object (−을/를, *−eul/leul*) differ from auxiliary particles, such as -은/는, *−eun/neun*. In particular, auxiliary particles that serve functions beyond topicalization may interfere with processing due to their multiple functions. For instance, the marker -*eun/neun* can be employed for emphasis, as in 그는 영어는 잘 한다 (*Neun yeong-eo-neun jal han-da*), which translates to ‘(He is not so good at other subjects, but) he is good at English.’ Another usage is to indicate contrast, as seen in 인생은 짧고 예술은 길다 (*In-saeng-eun jjalb-go ye-sul-eun gil-da*), meaning ‘Life is short, art is long.’ Additionally, it can function as a pseudo-subject particle in certain contexts where the subject has already been mentioned. In such cases, its function may not necessarily be as a topic marker. Additionally, the Korean language is also a highly contextual language. Depending on the use of the auxiliary particle, the nuance of a given sentence varies because it carries semantic information rather than the syntactic function. Thus, it is essential to be cautious of the multiple functions of the particle (marker) *-eun/neun* when interpreting the results of an experiment.

In verb-final languages such as Korean and Japanese, the sentence-ending verb also plays a crucial role in properly understanding a sentence, particularly when there is no animacy contrast between the subject and the object. This processing tendency can be described as a *backward argument-verb dependency* (Tamaoka and Mansbridge, 2019). In the absence of an animacy contrast, native Japanese speakers rely on information provided by the verb at the end of the sentence to establish the structural properties of scrambled constituents. Previous studies (Hsiao and Gibson, 2003; Pu, 2007; Vasishth et al., 2013; Kwon et al., 2019 for Mandarin Chinese; Mak et al., 2002 for Dutch) have indicated that animacy features affect the processing of relative clauses. Similarly, an animacy contrast may influence the processing of Korean and Japanese sentences, given that native speakers of these languages encounter the verb only at the end of the sentence. In such cases, animacy information might play a crucial role in constructing sentence structure, particularly in verb-final languages such as Korean and Japanese. Therefore, a semantically driven analysis for sentence processing would be considered an important additional factor.

It is widely accepted that both Korean and Japanese belong to the group of Altaic languages, sharing many linguistic features (Lee and Ramsey, 2000; Pae, 2024), although the debate over the designation of language family remains unsettled. In both Korean and Japanese, simple transitive sentences can exhibit either SOV or OSV word orders. In Japanese, noun phrases (NPs) are denoted by one of three case markers: the nominative -*ga* (NP_NOM_), the accusative -*o* (NP_ACC_), or the topicalization -*wa* (NP_TOP_). This system mirrors Korean in that the word orders demonstrate overlap in two primary aspects. First, sentences with a topicalized subject adhere to the same SOV structure as canonical sentences. Second, sentences where the object is topicalized align with the OSV order typical of scrambled sentences.

A study on topicalization in Japanese by Imamura et al. (2016) found the order of processing speed to be as follows: canonical S_NOM_O_ACC_V (M = 1,410 ms) = subject topicalized S_TOP_O_ACC_V (M = 1,414 ms) < scrambled O_ACC_S_NOM_V (M = 1,512 ms) < object topicalized O_TOP_S_NOM_V (M = 1,626 ms). This study showed that the processing time for the subject topicalized word order of S_TOP_O_ACC_V was the same as that of the canonical S_NOM_O_ACC_V. Therefore, as Imamura et al. (2016) suggested, this order seems to be commonly used, resulting in both structures being easily processed within a short time. A simpler explanation may be that when the nominative marker -*ga* is used, the topic marker -*wa* in the SOV order appears to identify the subject or its equivalent. Another possible explanation is that a sentence topic placed at the beginning of a sentence may speed up processing by initially providing an overall theme of the sentence. This discourse feature may explain equivalencies in processing speed. Additionally, it may be that a combination of these factors speeds up processing time.

However, the processing speed of object topicalized sentences was slower than their equivalent canonical sentences, and furthermore, even slower than their scrambled sentences. Thus, as Kuroda (1987) suggested, object topicalization could involve the process of both scrambling and topicalization. Additionally, it is possible to add the explanation that object topicalization focuses on ‘(an) apple’. This focus implies an exclusionary meaning of ‘an apple’ and ‘not any other fruits.’ Consequently, this focus may further delay the processing speed compared to corresponding scrambled sentences. Once again, because subject/object topicalization shares the same word order with canonical/scrambling, the effect of subject/object topicalization cannot be measured independently from canonical/scrambling.

### 1.2 Scrambling and topicalization in Tongan

In the Austronesian language of Tongan, the canonical word order is VSO (Verb-Subject-Object), commonly used in transitive sentences. However, a VOS (Verb-Object-Subject) order is also grammatically possible (Churchward, 1953; Dixon, 1979, 1994; Otsuka, 2000, 2005a,b; Custis, 2004). Tongan, being an ergative language (Otsuka, 2005a,b, 2010), marks both the subject of an intransitive sentence and the object of a transitive sentence with the same absolutive (ABS) marker ‘*a*, while the subject of a transitive sentence is marked by the ergative (ERG) marker ‘*e* (i.e., ERG/ABS case marking pattern). Tongan verbs have limited inflectional morphology (e.g., no inflection for tense). The present study focuses only on transitive sentences.

In Tongan, a noun phrase (NP) that is topicalized is positioned before the verb in either SVO or OVS orders, marked with the topic marker ‘*ko*’. This results in four potential word orders for transitive sentences: VSO, VOS, SVO, or OVS. Unlike in Japanese and Korean, when topicalization occurs in Tongan, the resulting word order does *not* overlap with either the VSO canonical or scrambled orders. To observe the topicalization effect as an independent phenomenon, the present study experimentally investigated processing times for these four orders of transitive sentences. A verb-initial language such as Tongan is ideal for investigating this processing function.

In Tongan, a sentence denoting ‘the woman ate the fish’ is assumed to be in the canonical order, as shown in Sentence (5), where NP refers to the noun phrase, PST refers to the past tense, ERG (‘*e*) refers to the ergative case marker, ABS (‘*a*) refers to the absolutive case marker, and REF refers to the referential/specific article (Anderson and Otsuka, 2006; Macdonald, 2014).

(5) VSO: Canonical transitive Tongan sentence.

*Na’e kai ‘e he fefine ‘a e ika*.

PST V(eat) ERG REF NP (woman) ABS REF NP (fish)

‘The woman ate the fish.’

The VOS scrambled order is derived from the VSO canonical order by moving the object (O) between the verb (V) and the subject (S), as shown in Sentence (6), constructing a more complex structure (VO_1_S*gap*_1_) than the canonical (VSO) order. The VOS scrambled order retains the same meaning of ‘The woman ate the fish.’

(6) VO_1_S*gap*_1_: Scrambled transitive Tongan sentence.

*Na’e kai ‘a e ika ‘e he fefine*.

PST V(eat) ABS REF NP (fish) ERG REF NP (woman)

‘The fish, the woman ate.’

According to the gap-filling parsing model (Frazier and Rayner, 1982; Stowe, 1986; Frazier, 1987; Frazier and Clifton, 1989; Frazier and Flores D’Arcais, 1989), native Tongan speakers possibly process VOS scrambled sentences in the following manner: The noun phrase ‘*a e ika* ‘the fish’ after the verb is an absolutive case marked (‘*a*) noun phrase indicating the object (O). Native Tongan speakers perceive that the object should come after the subject (S) to conform to the VSO canonical order. The object is temporarily kept in working memory (Chen, 1986; Carpenter and Just, 1989; King and Just, 1991) while the original object position of *gap*_1_ is identified as following S. Finally, speakers establish a relationship between O_1_ and *gap*_1_, and ‘the fish’ is filled into the *gap*_1_ position. The multiple steps required to use the active filler strategy should account for the extra processing time needed for the VOS scrambled order compared to the canonical VSO order.

In Tongan, there is a distinction in the topicalization of the subject and object. In the case of subject topicalization, a resumptive pronoun such as ‘ne’ (third person singular) appears in place of a gap in the relative clause (Otsuka, 2006), positioned after the past tense *na’a* (phonological change from *na’e*) and before the verb *kai* ‘eat’. In the English sentence, ‘This is the girl that I do not know what she said,’ ‘she’ in a relative clause refers to the previously mentioned noun ‘the girl’. Similarly, the resumptive pronoun *ne* in Tongan in Sentence (7) refers to *e fefine* ‘the woman’. In fact, the resumptive pronoun *ne* is necessary in the relativization of ergative NPs. Thus, topicalization behaves similarly to relativization in this aspect, indicating that it involves A-bar movement.

(7) S_1_V*gap*_1_O: Subject topicalized transitive Tongan sentence

*Ko e fefinei na’a nei kai ‘a e ika*

TOP REF NP (woman) PST PRO V(eat) ABS REF NP (fish)

‘(It is) the woman that (she) ate the fish.’

Custis (2004) posits that topicalization results from a movement marked by *ko*. Therefore, according to the gap-filling parsing model, a topicalized subject phrase is shifted in front of the verb. Native Tongan speakers identify the initial topicalized phrase as the filler and then seek its original position in the specifier of the gap to establish filler-gap dependency. As this dependency extends beyond the verb, processing an SVO subject topicalized sentence might require even more processing time than a VOS scrambled sentence. It is noteworthy that both subject and object topicalization in Tongan also convey an exclusionary meaning, similar to what is seen in Korean and Japanese.

The object can also be topicalized by moving it in front of the verb. As with subject topicalization, the object is also marked by the topic marker *ko*, as seen in *ko e ika* (‘the fish’) in Sentence (8), illustrating object topicalization (O_TOP_). However, unlike subject topicalization, object topicalization does not require a resumptive pronoun. Both Sentences (7 and 8) follow the structure *ko* NP_1_ V NP_2_. The object topicalization in Sentence (8) does not necessitate a resumptive pronoun.

(8) O_1_VS*gap*_1_: Object topicalized transitive Tongan sentence.

*Ko e ika na’e kai ‘e he fefine*.

TOP REF NP (fish) PST V(eat) ERG REF NP (woman).

‘About the fish, the woman ate.’

Object topicalization in Tongan may involve an even longer distance movement (O_1_VS*gap*_1_) than subject topicalization (S_1_V*gap*_1_O) because the object moves ahead of the subject and the verb. Native Tongan speakers may read a topicalized sentence to find *gap*_1_ at the end of the sentence as O_1_VS*gap*_1_. Once they establish the relationship between O_1_ and *gap*_1_, the topicalized O_1_ is filled in *gap*_1_. The distance between filler and gap in O_1_VS*gap*_1_ is longer than the distance between filler and gap in either VO_1_S*gap*_1_ or S_1_V*gap*_1_O. Therefore, based on the syntactic structure (moved distance), Tongan speakers keep O_1_ in working memory longer for O_1_VS*gap*_1_ than VO_1_S*gap*_1_ and S_1_V*gap*_1_O. Thus, the gap-filling parsing model predicts that an OVS topicalized order will take longer to process than either a VOS scrambled or SVO topicalized order. Additionally, if native Tongan speakers can obtain the argument information from the verb at the beginning of the sentence, they could easily construct a whole sentence based on that information. The processing of argument-verb dependency would function well to enable the construction of a whole syntactic structure of VSO and VOS. Since the subject and the object are placed before the verb in topicalization, native Tongan speakers may have to process the backward verb-argument dependency. In such a case, SVO and OVS orders may be disadvantaged in processing compared to VSO and VOS orders: a distinct difference in reaction times and possibly in accuracy may be observed between the verb-initial orders (VSO and VOS) and the verb-second orders (SVO and OVS).

From the perspective of syntactic complexity based on filler-gap dependency, processing difficulties among the four-word orders in Tongan sentences can be predicted as follows: The VS_ERG_ O_ABS_ canonical order is processed the fastest. In the V O_ABS1_ S_ERG_ *gap*_1_ structure, the object is moved in front of the subject, while in the topicalized S_TOP1_ V *gap*_1_ O_ABS_ sentence, the subject phrase is moved before the verb. Due to the distance of one phrase movement, the speed of sentence processing would be assumed to be similar between the scrambled VO_ABS1_ S_ERG_ *gap*_1_ and topicalized S_1_V*gap*_1_O orders. However, in object topicalization, the object phrase moves beyond both the verb and the subject phrases. Therefore, the O_TOP1_ V S_ERG_ *gap*_1_ object topicalized order has a much longer distance of filler-gap dependency, making it syntactically more complex than other word orders. This syntactically complex order would require the longest processing time among the four orders.

### 1.3 Outline of the present study

The present study conducted three experiments to investigate differences in word orders resulting from scrambling and topicalization. For native speakers, the accuracy of sentence correctness decisions tends to be consistently higher. This makes the reaction time required to determine whether a sentence is correct a more sensitive indicator. Therefore, sentence processing for various word orders can be predicted by processing speed.

Experiment 1 focused on (verb-final) Korean to compare the findings with those of Imamura et al. (2016) on Japanese. Given the syntactic similarities between Korean and Japanese, this experiment aimed to determine if a similar processing trend exists between the two languages. We anticipated that sentences in the S_NOM_ O_ACC_ V order would be processed most quickly because they are in the canonical order. Next, since topicalized sentences of S_TOP_ O_ACC_ V also have the same word order as the canonical order, we expected that they would be processed at the same speed as the canonical order, similar to Japanese sentences. Given that the scrambled order of O_ACC1_ S_NOM_ *gap*_1_V is known to be significantly slower than the canonical order, as shown by various studies (e.g., Mazuka et al., 2002; Ueno and Kluender, 2003; Koizumi and Tamaoka, 2004, 2010; Miyamoto and Takahashi, 2004; Tamaoka et al., 2005, 2014; Imamura et al., 2016; Witzel and Witzel, 2016; Tamaoka and Mansbridge, 2019), we expected O_TOP1_ S_NOM_ *gap*_1_V to be slower than both the canonical S_NOM_ O_ACC_ V and topicalized S_TOP_ O_ACC_ V orders. Furthermore, according to Imamura et al. (2016), the topicalized order of O_TOP1_ S_NOM_ *gap*_1_V was even slower than the scrambled order of O_ACC1_ S_NOM_ *gap*_1_V in Japanese, suggesting a similar result may be observed in Korean. Therefore, based on the filler-gap dependency (the movement-based anticipation), the speed of Korean sentence processing was predicted as follows:

**Prediction 1: Korean Sentence Processing Based on a Filler Gap Dependency.**

$$\begin{array}{l} S_{NOM}\;O_{ACC}\;V=\\ S_{\mathrm{TOP}}\;O_{ACC}\;V<O_{ACC1}\;S_{NOM}\;gap_{1}V<O_{\mathrm{TOP}1}\;S_{NOM}\;gap_{1}V\text{.} \end{array}$$

Experiment 2 involved a phrase-by-phrase processing experiment using a maze task to verify whether the VSO order is indeed canonical in the verb-initial language of Tongan. Similar to Experiment 1 in Korean, this experiment aimed to ascertain the canonical word order in Tongan sentence structure. Participants were presented with sentences having both VSO and VOS orders, broken down into phrases, and their processing speed for each phrase was measured. The results were then analyzed to determine whether the VSO order demonstrated superior processing efficiency compared to the VOS scrambled order, confirming its canonical status in Tongan sentence construction.

Experiment 3 in Tongan was conducted in a similar manner to Experiment 1 in Korean, enabling a comparative analysis of sentence processing strategies across the two languages. This experiment was designed to delve deeper into the effects of both scrambling and topicalization in Tongan sentence processing using a sentence correctness decision task. Specifically, it aimed to separate the influence of topicalization from the impact of canonical and scrambled orders. By comparing sentence processing across four different orders—VSO, VOS, SVO, and OVS—participants’ reaction times and accuracy rates were recorded and analyzed. This comprehensive investigation allowed for an understanding of how subject (SVO) and object topicalizations (OVS), in conjunction with canonical (VSO) and scrambled (VOS) orders, affect sentence processing in Tongan. Based on the filler gap dependency (movement-based anticipation), the speed of Tongan sentence processing was predicted as follows:

**Prediction 2: Tongan Sentence Processing Based on a Filler Gap Dependency.**

$$\begin{array}{l} V\;S_{\mathrm{ERG}}\;O_{\mathrm{ABS}}<V\;O_{\mathrm{ABS}1}\;S_{\mathrm{ERG}}\;gap_{1}=\\ S_{\mathrm{TOP}1}\;V\;gap_{1}\;O_{\mathrm{ABS}}<O_{\mathrm{TOP}1}\;V\;S_{\mathrm{ERG}}\;gap_{1}\text{.} \end{array}$$

The findings from these three experiments would contribute to our understanding of sentence processing mechanisms in both verb-final and verb-initial languages, shedding light on the universality and language-specific aspects of sentence structure and comprehension.

## 2 Experiment 1: processing of Korean scrambled and topicalized sentences

### 2.1 Method

#### 2.1.1 Participants

Forty-eight native Korean speakers (32 female and 16 male) were recruited from Busan National University in Korea. The mean age of the participants was 23.3 ± 2.6 years (range: 20–31 years). All collected information was stored in a secure location, and the participants were given numerical pseudonyms to ensure privacy. The present experiment involving human participants was reviewed and approved by the Research Ethics Committee of Busan National University. Participants signed informed consent forms before the experiment, and at the end of the experiment, they received payment and were debriefed.

#### 2.1.2 Stimulus sentences

Initially, 32 Korean SOV canonical sentences were created. Subsequently, 128 sentences corresponding to the four-word orders (32 × 4 = 128) were constructed following the format illustrated in Sentences (1) to (4). The complete list of stimulus sentences is provided in Supplementary material. As part of the control, 32 semantically incorrect sentences were formulated, ensuring that half of all sentences were semantically infelicitous and/or grammatically incorrect (hereafter referred to as ‘incorrect’). Each list thus consisted of 32 correct and 32 incorrect sentences, totaling 64 sentences. Examples of incorrect Korean sentences included 아이가 커튼을 헤엄쳤다 (*A-i-ga keo-teun-eul he-eom-chyeoss-da*), meaning ‘The child swam through the curtain,’ and 남동생이 사진을 운동했다 (*Nam-dong-saeng-i sa-jin-eul un-dong-haess-da*), meaning ‘My younger brother exercised a photo.’ The incorrect sentences are not included in Supplementary material. To ensure that the same sentence with different word orders would not be assigned to a single participant, these 128 sentences were counterbalanced into four lists, each to be distributed among the four participant groups.

#### 2.1.3 Procedure

**Figure 1:**
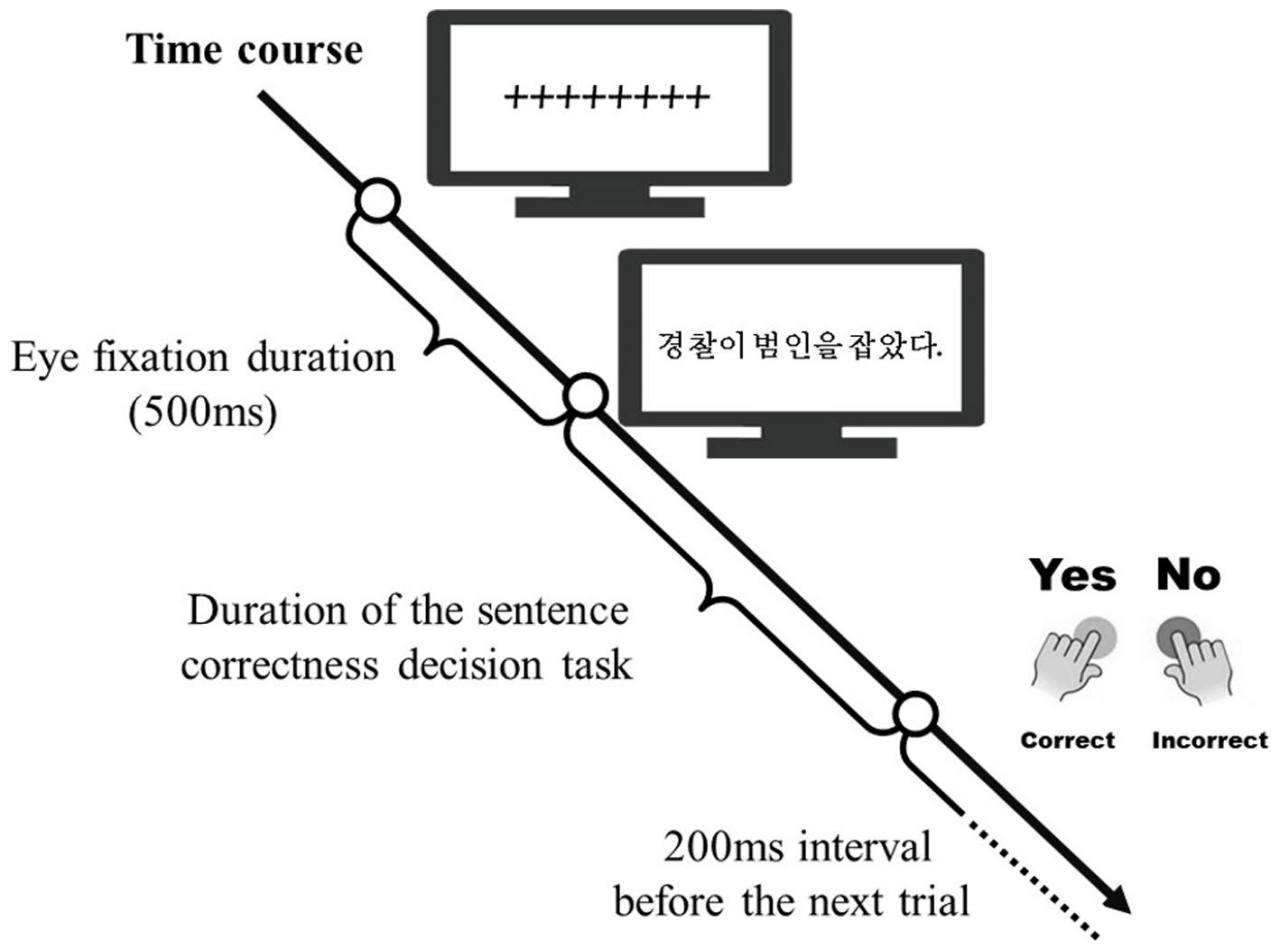
A single trial of the Korean sentence correctness decision task.

In Experiment 1, a Korean sentence correctness decision task was conducted on 48 native Korean speakers using their personal computers connected to the online experimental platform “Pavlovia”.^1^ As depicted in Figure 1, an eye fixation symbol (++++++++) was initially presented at the center of the computer screen for 500 ms, after which a target sentence replaced it. Participants were then required to decide whether the sentence was a correct Korean sentence (pressing the YES key for correct and the NO key for incorrect). The next trial appeared after a 200 ms interval. All stimulus sentences were randomly presented to each participant. Participants were instructed to complete the task as quickly and accurately as possible. Ten practice items were provided to each participant before the commencement of the actual experiment.

#### 2.1.4 Analysis

The accuracy and reaction times data collected from the sentence correctness decision task were analyzed using the linear mixed effect (LME) models (Baayen et al., 2008) and the *lme*4 package (Bates et al., 2016). The two fixed effects were trial and word order (four sentence conditions). The random effects were participants and stimulus sentences. The data for reaction times consisted only of data from trials with correct judgments. Satterthwaite’s approximations (Satterthwaite, 1946) were used via the *lmerTest* package to generate *p*-values for each model (Kuznetsova et al., 2017) using the restricted maximum likelihoods (Harville, 1977).

#### 2.1.5 Results of LME model analyses for accuracy data

A total of 1,536 responses (48 participants × 32 semantically and grammatically correct items) were analyzed. The fixed factors were trial and word order. The trial was centralized into *z*-values, coded as “trial.z.” The two random factors were participant and stimulus sentences. According to model comparisons using AIC (Anderson et al., 2000), the final best-fit LME model was *glmer*(acc ~ wordorder + trial.z + (0 + trial.z|participant) + (1|participant) + (1|item), data, family = binomial). The result of the best-fit LME model is reported in Table 1. The factor trial was significant [*z* = −3.22, *p* < 0.001]. This indicated that, as the experiment progressed, the accuracy of task performance decreased. The reference for word order was set as the canonical order of S_NOM_O_ACC_V. As shown in Table 1, the result indicated that S_NOM_O_ACC_V sentences were processed as accurately as S_TOP_ O_ACC_V topicalized sentences [*z* = −0.14, *ns*] but more accurately than O_ACC_ S_NOM_V scrambled sentences [*z* = −2.88, *p* < 0.01] and O_TOP_ S_NOM_V topicalized sentences [*z* = −3.53, *p* < 0.001].

**Table 1:** Result of the LME model analysis for accuracy.

Variables	Estimate	SE	*z* value	Pr(>|t|)	*p*-value
(Intercept)	5.11	0.57	9.03	*p* < 0.001	***
S_TOP_ O_ACC_V	−0.08	0.54	−0.14	*p* = 0.886	
O_ACC_ S_NOM_V	−1.33	0.46	−2.88	*p* < 0.01	**
O_TOP_ S_NOM_V	−1.59	0.45	−3.53	*p* < 0.001	***
trial.z	−0.62	0.19	−3.22	*p* < 0.001	***

Participants = 48. Item = 32. Total observation = 1,536. glmer(acc ~ wordorder + trial.z + (0 + trial.z|participant) + (1|participant) + (1|item), data, family = binomial).

**Figure 2:**
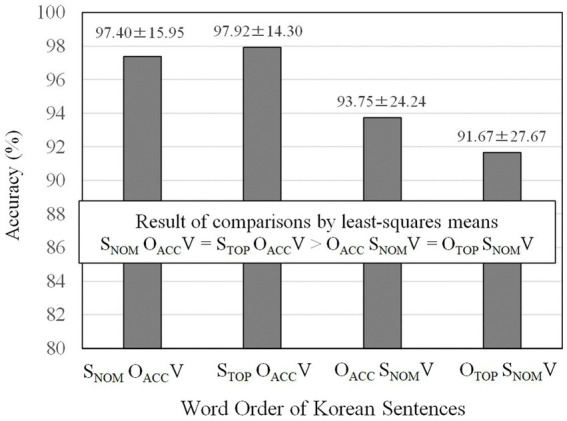
Accuracies of the four word orders of Korean sentences. The values after ± refer to standard errors.

To examine word order differences, accuracies of the four word orders were compared using the *lsmeans* (least-squares means; Searle et al., 1980) *R* package. The means and standard deviations are reported, and the result of multiple comparisons is shown in Figure 2. The result indicated that S_NOM_O_ACC_V (M = 97.40%) and S_TOP_O_ACC_V (M = 97.92%) were processed with the same level of accuracy. Both S_NOM_O_ACC_V and S_TOP_O_ACC_V orders were more accurately processed than both O_ACC_ S_NOM_V (M = 93.75%) and O_TOP_ S_NOM_V (M = 91.67%) orders. O_ACC_ S_NOM_V and O_TOP_ S_NOM_V orders were equally accurate.

#### 2.1.6 Results of LME model analyses for reaction time data

There were no stimulus sentences processed faster than 500 ms or slower than 6,000 ms. After removing 74 incorrectly answered items from the 1,536 semantically and grammatically correct items, the remaining 1,462 correctly answered items were analyzed on reaction times. Based on the Box-Cox power transformation technique (Box and Cox, 1964; Venables and Ripley, 2002), a logarithmic transformation (natural log) was applied to the reaction times to attenuate any skewness in their distribution. Reaction times were analyzed with the *lmer* function using the restricted maximum likelihood (Harville, 1977). Satterthwaite’s approximations (Satterthwaite, 1946) were used via the *lmerTest* package to generate *p*-values for each model (Kuznetsova et al., 2017).

According to model comparisons using AIC (Anderson et al., 2000), the best-fit LME model was *lmer* (log(rt) ~ wordorder + trial.z + (1|participant) + (1|item), data). Based on this best-fit LME model, potentially influential outliers with absolute standardized residuals exceeding 2.5 standard deviation were removed. Of the 1,462 responses, 34 responses were removed. The result of the LME model analysis for the 1,428 responses is reported in Table 2. Unlike for accuracy, the trial was not a significant factor [*t*(1104.00) = −0.34, *ns*]. The reference for word order was set as S_NOM_O_ACC_V. As shown in Table 2, this result indicated that S_NOM_O_ACC_V order was processed at the same speed as S_TOP_ O_ACC_V topicalized order [*t*(1346.00) = −0.09, *ns*], but faster than O_ACC_ S_NOM_V scrambled order [*t*(1355.00) = 8.64, *p* < 0.001] and O_TOP_ S_NOM_V topicalized order [*t*(1355.00) = 9.79, *p* < 0.001].

**Table 2:** Result of the LME model analysis for reaction times.

Variables	Estimate	SE	df	*t* value	Pr(>|t|)	*p*-value
(Intercept)	0.17	0.04	78.36	4.02	*p* < 0.001	***
S_TOP_ O_ACC_V	−0.002	0.02	1,346.00	−0.09	*p* = 0.927	
O_ACC_ S_NOM_V	0.19	0.02	1,355.00	8.64	*p* < 0.001	***
O_TOP_ S_NOM_V	0.21	0.02	1,355.00	9.79	*p* < 0.001	***
trial.z	−0.003	0.01	1,104.00	−0.34	*p* = 0.733	

Participants = 48. Item = 32. Total observation = 1,428 responses (74 incorrectly answered responses and 34 outliers were removed from the total observation of 1,536 responses). lmer(log(rt) ~ wordorder + trial.z + (1|participant) + (1|item), data).

**Figure 3:**
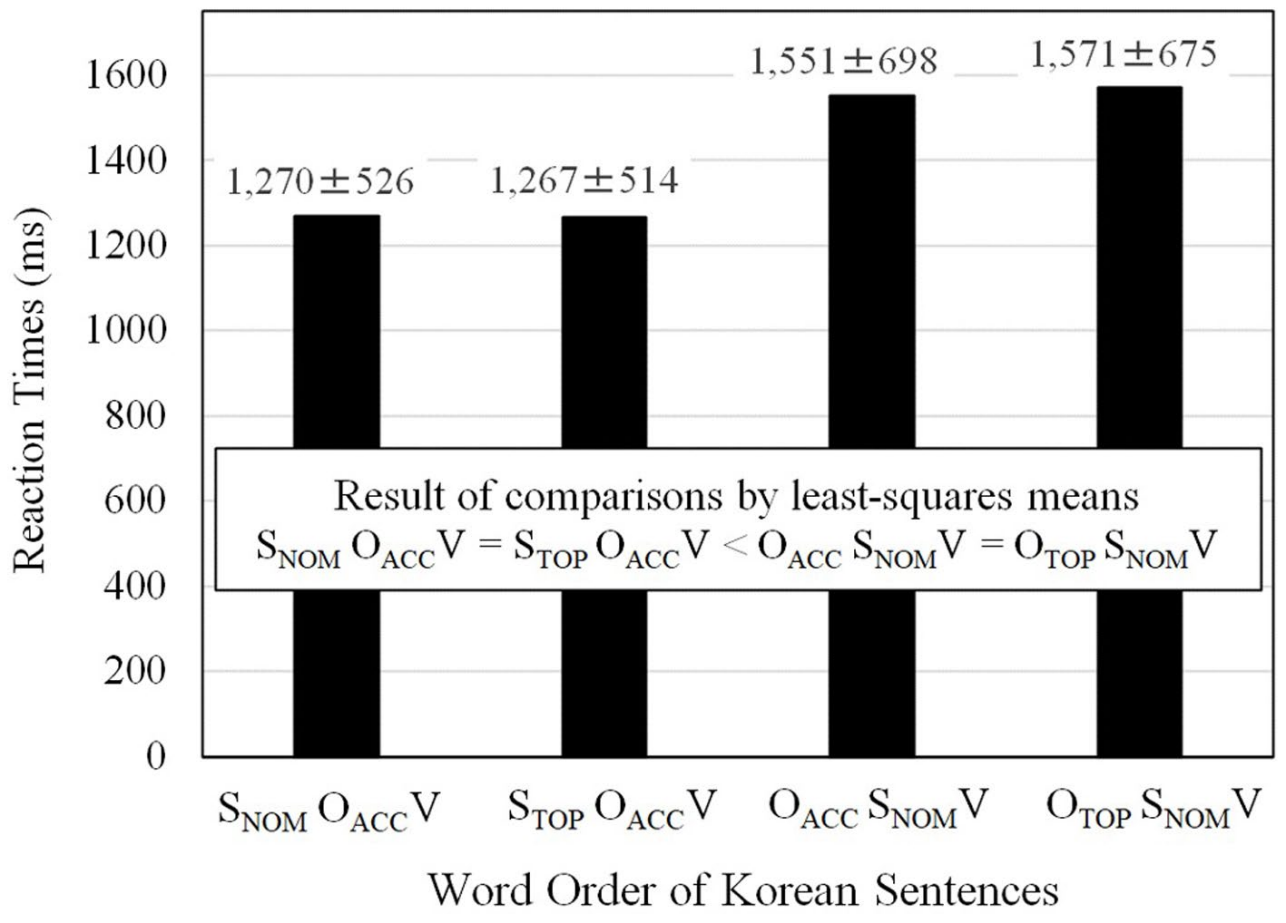
Reaction times of the four word orders of Korean sentences. The values after ± refer to standard errors.

For a detailed examination of the differences in reaction times among word orders, the times for the four word orders were compared using the *R* package of *lsmeans* (least-squares means; Searle et al., 1980). The means and standard deviations (1,428 responses) were reported, and the result of multiple comparisons is shown in Figure 3. The result indicated that S_NOM_O_ACC_V (M = 1,270 ms) and S_TOP_O_ACC_V (M = 1,267 ms) orders were processed at the same speed. Both S_NOM_O_ACC_V and S_TOP_O_ACC_V orders were processed faster than both O_ACC_ S_NOM_V (M = 1,551 ms) and O_TOP_ S_NOM_V (M = 1,571 ms) orders. O_ACC_ S_NOM_V and O_TOP_ S_NOM_V orders were processed at roughly the same speed.

### 2.2 Discussion

The results of the Korean sentence correctness decision task from Experiment 1 demonstrated a pattern similar to that observed in the study by Imamura et al. (2016) in Japanese, largely confirming Prediction 1, with the exception of no difference observed between O_ACC_ S_NOM_V and O_TOP_ S_NOM_V. First, akin to Japanese, the scrambled order in Korean was processed more slowly than the canonical order. Second, consistent with the findings of the previous Japanese experiment (Imamura et al., 2016), the subject topicalized order was processed at a comparable speed to the canonical order. This observation suggests that similar to Japanese, the S_TOP_O_ACC_V subject topicalized order in Korean might have been construed simply as the subject, akin to the canonical ordered sentence S_NOM_O_ACC_V. Third, unlike Japanese, there was no discernible difference in processing time between object topicalized and scrambled sentences. This discrepancy between Japanese and Korean could be attributed to the animacy effect, as Korean stimulus sentences featured animacy contrast, unlike the study by Imamura et al. (2016). Further elaboration on this aspect will be provided in the General Discussion section. Additionally, similar to Japanese, the SOV canonical order in Korean overlapped with the subject topicalized order, while the OSV scrambled order in Korean overlapped with the object topicalized order. Consequently, neither Japanese nor Korean could distinguish between the effects of scrambling and topicalization. Hence, a more comprehensive investigation is warranted in Tongan, which exhibits a distinct word order for canonical/scrambled and subject/object topicalization.

## 3 Experiment 2: verification of the VSO canonical order in Tongan

This experiment aimed to verify whether the VSO order is indeed canonical in sentence processing by native Tongan speakers.

### 3.1 Methods

#### 3.1.1 Participants

Forty-eight native Tongan speakers (33 female and 15 male) were recruited from the Tonga Institute of Education located on the main island of Tongatapu, Tonga. The mean age of the participants was 22.8 ± 4.6 years (range 17–35 years). All participants received monetary compensation in exchange for their participation and provided written informed consent. The present experiment involving human participants was reviewed and approved by the Research Ethics Committee of Tohoku University. All collected information was stored in a secure location, and the participants were given numerical pseudonyms to ensure privacy. The participants signed informed consent forms before the experiment, and at the end of the experiment, they received payment and were debriefed.

Both Tongan and English are the official languages in Tonga. As a result of globalization, English is frequently used in Tonga (Otsuka, 2007). However, in daily life, native Tongans use Tongan more frequently than English; therefore, Tongan is considered to be their first language. To analyze how native Tongans use the two languages, the present study conducted a questionnaire survey on 48 participants regarding their use of the two languages and their perceptions of their own language proficiencies. The survey found that the mean use percentage of Tongan in daily life was 79.98% (SD 15.35%), whereas the mean use for English was 23.92% (SD 15.26%). It should be noted that a question for Tongan or English daily use was asked independently, so usage percentages of Tongan and English do not perfectly sum up to 100% (79.98% + 23.92% = 103.9%). This 64.63% usage difference between Tongan and English was significant [*F*(1, 47) = 138.91, *p* < 0.001, *η_p_*^2^ = 0.75]. Subjective proficiency judgments of four important language skills (speaking, listening, reading, and writing) for Tongan and English were measured using a 0-to-6 point scale (0 ‘none’ to 6 ‘very high’): Speaking skills between Tongan (M = 5.60, SD = 0.64) and English (M = 4.38, SD = 0.91) differed significantly [*F*(1, 47) = 62.57, *p* < 0.001, *η_p_*^2^ = 0.57]; listening skills between Tongan (M = 5.50, SD = 0.74) and English (M = 4.58, SD = 1.09) differed significantly [*F*(1, 47) = 38.17, *p* < 0.001, *η_p_*^2^ = 0.45]; reading skills between Tongan (M = 5.56, SD = 0.60) and English (M = 4.81, SD = 0.98) differed significantly [*F*(1, 47) = 31.67, *p* < 0.001, *η_p_*^2^ = 0.40]; finally, writing skills between Tongan (M = 5.31, SD = 0.78) and English (M = 4.60, SD = 1.11) also differed significantly [*F*(1, 47) = 14.16, *p* < 0.001, *η_p_*^2^ = 0.23]. In summary, both indexes of language use percentages and subjective language skill judgments indicated a high level of proficiency in Tongan and good proficiency (but to a lesser degree than Tongan) in English.

#### 3.1.2 Stimulus sentences

Thirty transitive sentences were composed. Each sentence contained four phrases, including an initially presented adverb (*Adv*) *mahalo* ‘maybe’, a verb (V), and two noun phrases. The frequently used first names of *Taniela* and *Kaufusi* were used in all the “two noun” phrases. An example of this is the sentence, *Na‘e talitali ‘e Taniela ‘a Kaufusi* meaning ‘Maybe Taniela welcomed Kaufusi’. Based on the canonical order of *Adv*VSO illustrated in Sentence (9), the 30 *Adv*VOS ordered scrambled sentences were created by exchanging the absolutive marker ‘*a* and ergative marker ‘*e* for the two first names, resulting in an *Avd*VOS scrambled order as seen in Sentence (10).

(9) *Adv*VSO canonical order.

Phrase 1 Phrase 2 Phrase 3 Phrase 4.

*Mahalo na‘e talitali ‘e Taniela ‘a Kaufusi*.

*Adv*(maybe) V(welcome)-PAST NP-ERG (Taniela) NP-ABS (Kaufusi).

‘Maybe Taniela welcomed Kaufusi.’

(10) *Adv*VOS scrambled order.

Phrase 1 Phrase 2 Phrase 3 Phrase 4.

*Mahalo na‘e talitali ‘a Taniela ‘e Kaufusi*.

*Adv*(maybe) V(welcome)-PAST NP-ABS (Taniela) NP-ERG (Kaufusi).

‘Maybe Kaufusi welcomed Taniela.’

As shown in Sentences (9) and (10), the first names “*Taniela* and *Kaufusi*” were kept in the same position within the sentence. Through this manipulation, the differences in each region of the two case markers for the noun phrases could be directly compared using the maze task (Forster et al., 2009; Forster, 2010, the method is explained in the following section). The experimental stimuli (correct lexical sentences) are listed in Supplementary material. Incorrect lexical items are not included in Supplementary material. The stimuli were counterbalanced to ensure that participants would see only one format for each sentence per experimental session.

#### 3.1.3 Measuring reaction times with the lexical maze task

The present study employed a measurement tool for sentence processing known as the “maze task” (Forster et al., 2009 for English; Witzel and Witzel, 2016 for Japanese; and Qiao et al., 2012 for Mandarin Chinese). This experiment was conducted individually by a native Tongan speaker in a classroom at the Tonga Institute of Education.

**Figure 4:**
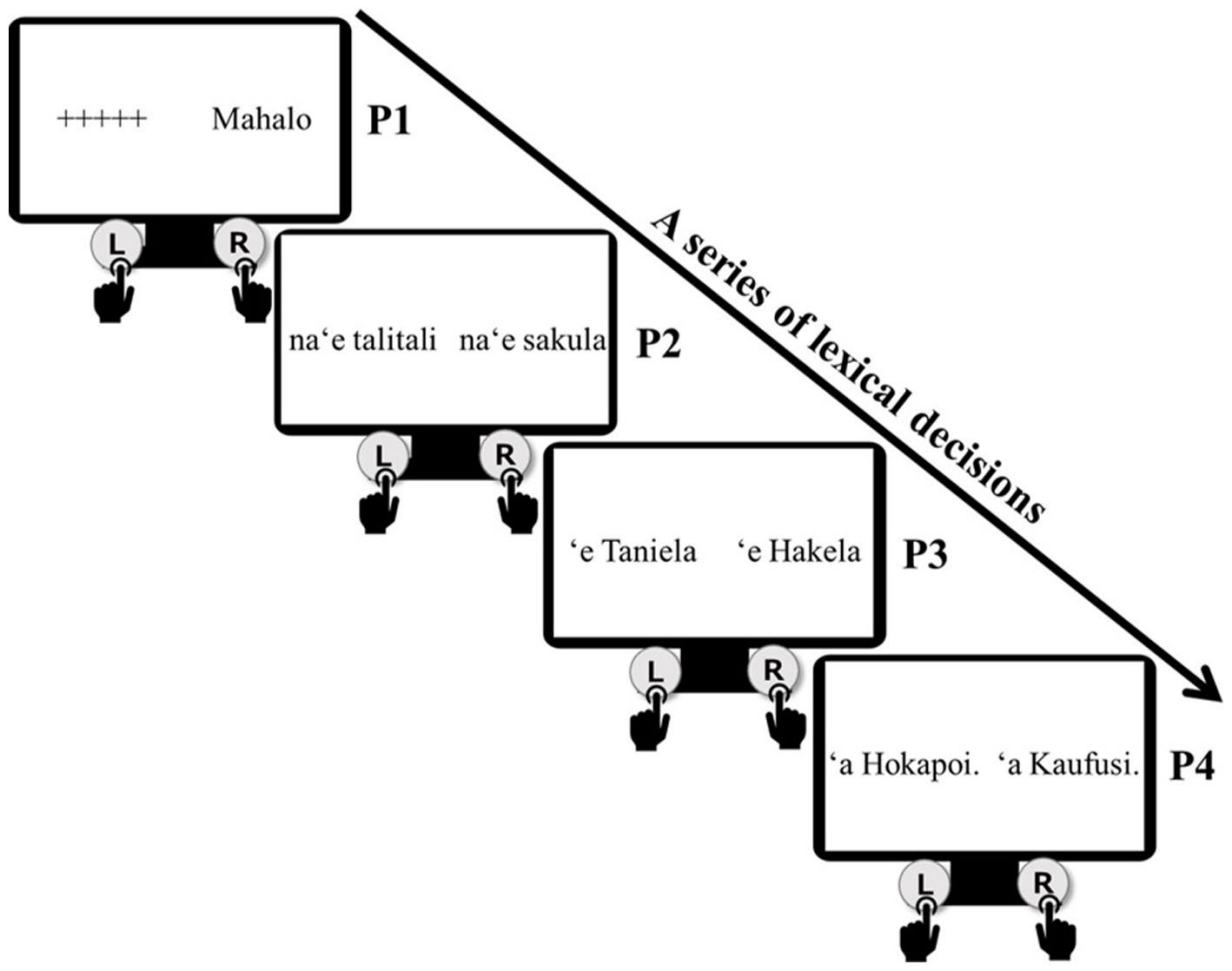
A series of lexical decisions in the lexical maze task.

In this experiment, both a real word and a non-word are simultaneously presented on the left and right sides of a computer screen. As shown in Figure 4, initially ‘+++++’ and *Mahalo* (Maybe) with a capitalized first letter were presented. Participants were instructed to choose *Mahalo* by pressing the right key. This was the initial lexical decision across all trials. After 200 ms, *na‘e talitali* (‘welcomed’; a real word) and *na‘e sakula* (non-word) were presented. The participants were directed to select the real word in Tongan. The past tense of *na‘e* was kept as a constant, so that participants would make a lexical decision based only on their assessment of the verb form of *talitali* ‘welcome’. In this case, a correct decision would have been made by pressing the left key. After another 200 ms, the two first names of *Taniela* and *Hakela*, both with the absolutive case marker ‘*e*, were presented. The first name *Taniela* is a real name in Tongan, while *Hakela* is not, so participants were expected to press the left key. Finally, after a further 200 ms, *Hokapoi* and *Kaufusi*, both with the ergative case maker ‘*a*, were presented. The first name *Kaufusi* is the real name, so the participant was expected to press the right key. As the case markers of the third (P3) and fourth (P4) phrases were kept the same on both the left and right sides, the participant had to decide whether these were correct expressions in Tongan solely by focusing on the first name. Within the flow of lexical decision-making, the series of correct real words constructed a four-phrase real sentence, i.e., the *Avd*VSO canonical ordered sentence, *Mahalo na‘e talitali ‘a Kaufusi* ‘Maybe Taniela welcomed Kaufusi’ P1 to P4 in Figure 4 illustrates the series.

If the participant made a mistake before the final phrasal set, the trial was stopped, and the next trial started 600 ms later. Two stimulus phrases were randomly positioned on the left and right sides of the screen in each trial. The participants were asked to perform the maze task as quickly and accurately as possible. Before the actual experiment began, eight practice trials were given.

#### 3.1.4 Results

##### 3.1.4.1 Data from the lexical maze task

The reaction times for the lexical maze task were analyzed using a linear mixed effect (LME) model (Baayen et al., 2008) and the *lme*4 package (Bates et al., 2016). For every analysis of the four phrases of P1 to P4, the fixed variables were phrasal order (*Adv*VSO canonical order versus *Adv*VOS scrambled order) and trial (centralized as *z*-vales). The random variables were participants and stimulus sentences. In the lexical maze task, any mistake made during the performance terminated the processing. There were 109 incorrect responses out of 1,440 total trials (92.43% correct rate or 7.57% incorrect rate), meaning that 1,331 correct responses were used for analysis. The reaction times of the four phrases were analyzed.

##### 3.1.4.2 Results of LME model analyses

**Figure 5:**
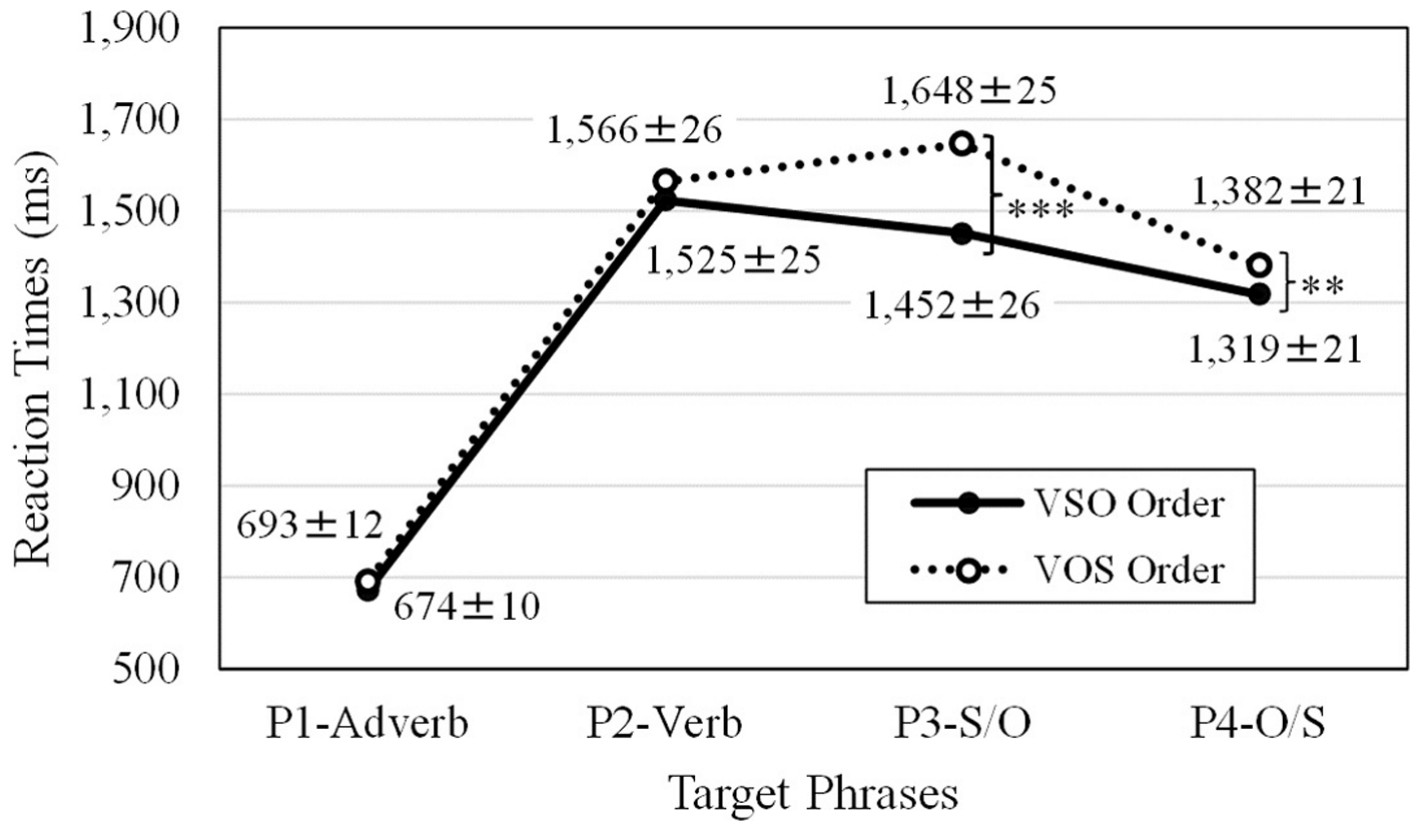
Reaction times for processing first phrase (P1) to fourth phrase (P4) of canonical *Adv*VSO and scrambled *Adv*VOS ordered sentences. The values after ± refer to standard errors.

The Box-Cox power transformation technique (Box and Cox, 1964; Venables and Ripley, 2002) indicated that a reciprocal transformation (−1,000/rt; rt. referring to reaction times) for the first phrase (P1) and a square root transformation for the second phrase (P2) to the fourth phrase (P4) was applied to the reaction time data to attenuate skewness in the distribution. Reaction times were analyzed with the *lmer* function with the restricted maximum likelihood (Harville, 1977). Satterthwaite’s approximations (Satterthwaite, 1946) were used via the *lmerTest* package to generate *p*-values for each model (Kuznetsova et al., 2017). After that, the best-fit LME model was found based on model comparisons using AIC (Akaike’s Information Criterion compared by the maximum likelihood; Anderson et al., 2000). Outliers with absolute standardized residuals exceeding 2.5 standard deviations were removed, and then the same best-fit LME model was reapplied to the resulting data set. This LME analysis procedure was repeated for the reaction times from the first phrase (P1-Adverb) to the fourth phrase (P4-O/S). The means and standard errors from all phases are depicted in Figure 5.

LME analysis was conducted on reaction times for the first phrase (P1-Adverb). After 26 responses (1.95%) were removed from 1,331 total correct responses, 1,305 responses were used for the final analysis. LME command was *lmer* (−1,000/rt ~ (0 + trial.z|participant) + (1|participant) + (1|item) + wordorder + trial.z, data). The trial order was centralized as *z* values (trail.z). Reaction times for the first phrase with the adverb *Mahalo* (P1-Adverb) showed a significant trial order effect [*t*(45.52) = −4.23, *p* < 0.001], but no significant difference [*t*(1215.31) = −1.16, *ns*] between the canonical *Adv*VSO (M = 674 ms, SD = 257 ms, SE = 10 ms) and scrambled *Adv*VOS orders (M = 693 ms, SD = 302 ms, SE = 12 ms). Since the same adverb *Mahalo* was presented in both the canonical and scrambled orders, no differences were expected.

Similarly, an LME analysis was conducted on reaction times for the second phrase (P2-Verb). After 32 responses (2.40%) were removed from 1,331 total correct responses, 1,299 responses were used for the final analysis. The trial order was centralized as *z* values (trail.z). LME command was *lmer* (sq_rt2 ~ wordorder + trial.z + (0 + trial.z|subject) + (1|subject) + (1|item), data). Reaction times for the second phrase of the verb (P2-Verb) showed a significant trial order effect [*t*(46.65) = −2.52, *p* < 0.05], but, once again, there were no significant differences in word order [*t*(1203.92) = −1.08, *ns*]. Because the same verbs were presented as the correct choice for the lexical decisions in both the canonical *Adv*VSO (M = 1,525 ms, SD = 647 ms, SE = 25 ms) and scrambled *Adv*VOS (M = 1,566 ms, SD = 648 ms, SE = 26 ms) orders, a null result for order was also expected for this phrase of the sentence.

As shown in Table 3, reaction times for the third S/O phrase (P3-S/O) showed a significant difference between recognizing the subject for the canonical *Adv*VSO (M = 1,452 ms, SD = 657, SE = 26) order and the object for scrambled *Adv*VOS (M = 1,648 ms, SD = 644 ms, SE = 25) order [*t*(1212.90) = −7.42, *p* < 0.001], as well as for the trial order effect [*t*(45.55) = −4.42, *p* < 0.001]. A noun phrase with the ergative marker ‘*e* (subject) would be expected to come after the verb in the canonical VSO order in Tongan. This result supports the linguistic proposal that VSO is the canonical order in Tongan.

**Table 3:** Result of the LME analysis for reaction times for the third phrase (P3-S/O).

Variables	Estimate	SE	df	*t* value	Pr(>|t|)	*p*-value
(Intercept)	40.01	0.57	75.51	70.70	*p* < 0.001	***
Phrasal order	−2.37	0.32	1212.90	−7.42	*p* < 0.001	***
trial.z	−1.07	0.24	45.55	−4.42	*p* < 0.001	***

lmer (sq_rt3 ~ wordorder + trial.z + (0 + trial.z|participant) + (1|participant) + (1|item), data). After 28 responses (2.10%) were removed from 1,331 total correct responses, 1,303 responses were used for the final analysis. The trial order was centralized as *z* values (trail.z).

As shown in Table 4, reaction times for the fourth O/S phrase (P4-O/S) showed a significant difference between recognizing the object for the canonical *Adv*VSO (M = 1,319 ms, SD = 540 ms, SE = 21 ms) order and the subject for the scrambled *Adv*VOS (M = 1,382 ms, SD = 530 ms, SE = 21 ms) order [*t*(1203.97) = −2.60, *p* < 0.01] as well as for the trial order effect [*t*(47.05) = −6.11, *p* < 0.001]. Noun phrases with the absolutive marker ‘*a* (object) were processed faster than noun phrases with the ergative marker ‘*e* (subject), even though the final phrase would automatically be understood to include the object for the canonical and the subject for the scrambled order. This difference in the final fourth phase further supports the VSO canonical order advantage against its VOS scrambled counterpart.

**Table 4:** Result of the LME analysis for reaction times for the fourth phrase (P4-O/S).

Variables	Estimate	SE	df	*t* value	Pr(>|t|)	*p*-value
(Intercept)	36.60	0.67	75.93	54.90	*p* < 0.001	***
Phrasal order	−0.78	0.30	1203.97	−2.60	*p* < 0.01	**
trial.z	−1.28	0.21	47.05	−6.11	*p* < 0.001	***

lmer (sq_rt4 ~ wordorder + trial.z + (0 + trial.z|participant) + (1|participant) + (1|item), data). After 34 responses (2.55%) were removed from 1,331 total correct responses, 1,297 responses were used for the final analysis. The trial order was centralized as z values (trial.z).

### 3.2 Discussion

Experiment 2 utilized a maze task to assess the processing of Tongan *Adv*VSO canonical and *Adv*VOS scrambled orders phrase-by-phrase. The results concerning the third and fourth crucial phrases occurring after the verb indicated that native Tongan speakers process the VSO order faster than the VOS order. Consistent with previous linguistic studies (Churchward, 1953; Dixon, 1979, 1994; Otsuka, 2000, 2005a,b, 2010), the present psycholinguistic investigation confirmed that the VSO order is canonical in Tongan. Experiment 2 provided support for the scrambling effect (V S_ERG_ O_ABS_ < V O_ABS1_ S_ERG_ *gap*_1_) as depicted in Prediction 2 based on filler-gap dependency.

## 4 Experiment 3: observing topicalization and scrambling effects independently

Building upon the findings of Experiment 2, which demonstrated that the canonical order in Tongan is VSO and the scrambled order is VOS, Experiment 3 aimed to conduct a comprehensive investigation into the effects of canonical/scrambled word orders and subject/object topicalization. Specifically, this experiment sought to assess the processing efficiency, including both speed and accuracy, of the four word orders (VSO, VOS, SVO, and OVS) in Tongan sentences.

### 4.1 Methods

#### 4.1.1 Participants

Forty native Tongan speakers (28 female and 12 male) were recruited on the main island of Tonga (Tongatapu) by a native Tongan experimenter hired to conduct Experiment 3. The mean age of the participants was 29.3 ± 6.4 years (range: 21–38 years). All participants received monetary compensation in exchange for their participation and provided written informed consent. The present experiment involving human participants was reviewed and approved by the Research Ethics Committee of Tohoku University. All collected information was stored in a secure location, and the participants were given numerical pseudonyms to ensure privacy. Participants from Experiment 2 did not participate in Experiment 3. As indicated by the result of the questionnaire survey in Experiment 2 (see ‘participants’ section in Experiment 2), Tongatapu residents are highly proficient in Tongan as their first language and also have good proficiency in English as their second language.

#### 4.1.2 Stimulus sentences

Thirty-two VSO canonical, VOS scrambled, SVO subject topicalized, and OVS object topicalized sentence pairs (32 × 4 = 128 items) were created as shown in example Sentences (5) to (8). All the semantically and grammatically correct sentences are listed in Supplementary material. Each sentence consisted of three phrases consisting of a transitive verb and two noun phrases. Transitive verbs were all expressed in the past tense *na‘e*. Frequently used first names or human nouns were used as the subject with the ergative case marker ‘*e*, whereas the frequently used inanimate or non-human nouns with the absolutive case marker ‘*a* were used as the object. When the object was topicalized, the topicalization maker *ko* was used. In sentences in which a first name was not used, all animate and inanimate nouns were marked by the definite article *e/he*. The 128 experimental sentences were counterbalanced to ensure that participants would see only one format of each sentence per experimental session.

An equal number of 32 semantically and/or grammatically incorrect sentences were also created, comprising 8 VSO, 8 VOS, 8 SVO, and 8 OVS-ordered sentences (32 in total). An example of an incorrectly ordered VSO sentence is *Na′e inu ‘e he tahi ‘a e vaka*, meaning ‘The sea drank the boat.’ Since incorrect responses were not used for analysis, only one set was prepared with no counterbalance. Additionally, 10 correct and 10 incorrect sentences unrelated to the present study were included. These 20 dummy sentences were also not used for analysis. Therefore, each participant received a total of 84 sentences: (1) counterbalanced sentences of VSO, VOS, SVO, and OVS ordered correct sentences (*N* = 8 each), (2) non-counterbalanced VSO, VOS, SVO, and OVS ordered incorrect sentences (*N* = 8 each), and (3) 20 dummy sentences consisting of 10 correct and 10 incorrect sentences. An equal proportion of 42 correct sentences and 42 incorrect sentences were used to ensure the same number of correct and incorrect stimuli. The stimulus sentences listed in Supplementary material do not include the incorrect sentences or dummy sentences.

#### 4.1.3 Procedure

As in Experiment 1, Experiment 3 employed a sentence correctness decision task. The eye fixation of ‘++++++++’ was initially presented at the center of the computer screen for 500 ms, and then replaced by a target sentence. Participants were required to decide whether the presented sentence was grammatically and semantically correct in Tongan by pressing the YES key for correct or the NO key for incorrect. After pressing either key, the next trial started after 200 ms. The participants were asked to perform the sentence correctness decision task as quickly and accurately as possible. All stimulus sentences were randomly presented for each participant. Before the experiment started, 10 practice sentences were given. Experiment 3 was conducted individually face-to-face in a quiet room by a native Tongan experimenter using her own computer connected to the online experimental research environment “Pavlovia” (see footnote 1).

#### 4.1.4 Results

##### 4.1.4.1 Analysis

The same LME analyses used in Experiment 1 were applied in Experiment 3.

##### 4.1.4.2 Results of LME model analyses for accuracy data

A total of 1,280 responses (40 participants × 32 semantically and grammatically correct items) were analyzed. The fixed factors were trial and word order. The trial was centralized into *z*-values, coded as trial.z. The two random factors were participants and stimulus sentences. According to model comparisons using AIC (Anderson et al., 2000), the final best-fit LME model was *glmer*(acc ~ wordorder + trial.z + (1|participant) + (1|item), data, family = binomial). The result of the best-fit LME model is reported in Table 5. Trial was not a significant factor [*z* = −0.62, *ns*]. Based on the reference of VS_ERG_O_ABS_ canonical order, the result in Table 5 indicated that VS_ERG_O_ABS_ canonical sentences were processed as accurately as O_TOP_VS_ERG_ object topicalized sentences [*z* = −1.35, *ns*], but more accurately than VO_ABS_S_ERG_ scrambled sentences [*z* = −2.80, *p* < 0.01] and S_TOP_VO_ABS_V subject topicalized sentences [*z* = −2.29, *p* < 0.05].

**Table 5:** Result of the LME model analysis for accuracy.

Variables	Estimate	SE	*z* value	Pr(>|t|)	*p*-value
(Intercept)	6.13	0.89	6.86	*p* < 0.001	***
VO_ABS_ S_ERG_	−2.17	0.77	−2.80	*p* < 0.01	**
S_TOP_VO_ABS_	−1.81	0.79	−2.29	*p* < 0.05	*
O_TOP_VS_ERG_	−1.13	0.84	−1.35	*p* = 0.178	
trial.z	−0.12	0.19	−0.62	*p* = 0.533	

Participants = 40. Items = 32. Total Observations = 1,280. glmer(acc ~ wordorder + trial.z + (1|participant) + (1|item), data, family = binomial).

**Figure 6:**
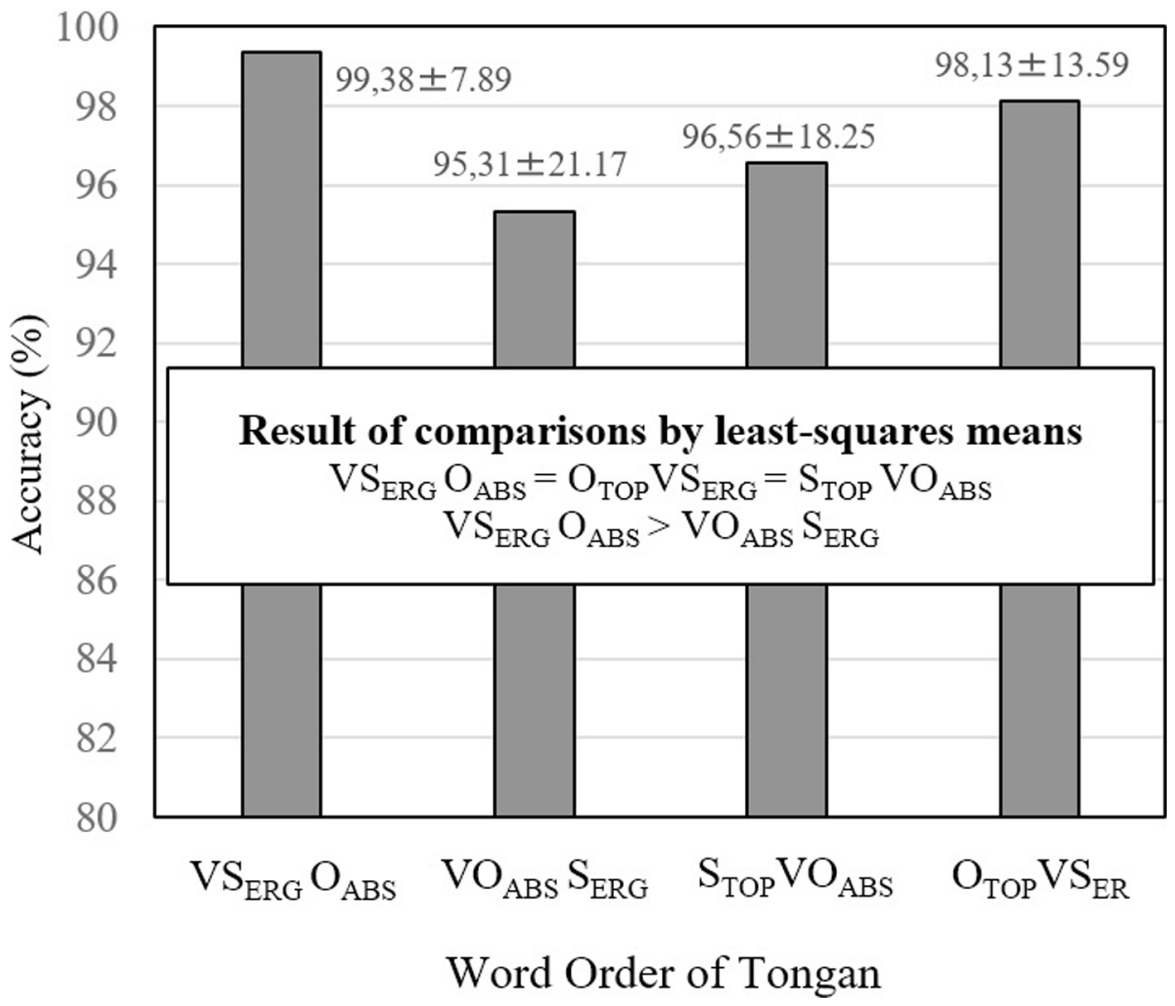
Accuracies for the four word orders of Tongan sentences. The values after ± refer to standard errors.

To examine differences among word orders more in depth, the accuracies of the four sentence conditions were compared using the *R* package of *lsmeans* (Searle et al., 1980). The means and standard deviations are reported, and the results of multiple comparisons are shown in Figure 6. The result indicated that VS_ERG_O_ABS_ (M = 99.38%), S_TOP_VO_ABS_ (M = 96.56%), and O_TOP_VS_ERG_ (M = 98.13%) were processed equally accurately. Both subject and object topicalized sentences were processed as accurately as canonical sentences. By contrast, VS_ERG_O_ABS_ canonical sentences were processed more accurately than their corresponding VO_ABS_S_ERG_ scrambled sentences (M = 95.31%). This result illustrates the scrambling effect. Since the multiple comparisons by *lsmeans* did not show a significant difference in results for VS_ERG_O_ABS_ and S_TOP_VO_ABS_, we interpret the difference shown in Table 5 of the LME analysis as a significant tendency rather than as a significant difference.

##### 4.1.4.3 Results of LME model analyses for reaction time data

There were no items processed faster than 500 ms or more slowly than 5,000 ms. After removing 34 incorrectly answered items from the 1,280 semantically and grammatically correct items, the remaining 1,246 correctly answered items were analyzed for reaction times. Based on the Box-Cox power transformation technique (Box and Cox, 1964; Venables and Ripley, 2002), a logarithmic transformation (natural log) was applied to the reaction times to attenuate skewness in their distribution. Reaction times were analyzed with the *lmer* function using the restricted maximum likelihood (Harville, 1977). Satterthwaite’s approximations (Satterthwaite, 1946) were used via the *lmerTest* package to generate *p*-values for each model (Kuznetsova et al., 2017).

According to model comparisons using AIC (Anderson et al., 2000), the best-fit LME model was *lmer* (log(rt) ~ wordorder + trial.z + (0 + trial.z|participant) + (1|participant) + (1|item), data). Based on this best-fit LME model, potentially influential outliers with absolute standardized residuals exceeding 2.5 standard deviation were removed. In this operation, 28 responses were removed. The result of the LME model analysis for 1,218 responses is reported in Table 6. As with accuracy, trial was not a significant factor [*t*(50.06) = −0.85, *ns*]. Based on the reference canonical order of VS_ERG_O_ABS_V, the result in Table 6 indicated that VS_ERG_O_ABS_ canonical sentences were processed equally as quickly as O_TOP_VS_ERG_V object topicalized sentences [*t*(1131.53) = 1.93, *ns*] but more quickly than VO_ABS_S_ERG_ scrambled sentences [*t*(1127.06) = 5.53, *p* < 0.001] and S_TOP_VO_ABS_ subject topicalized sentences [*t*(1122.14) = 2.77, *p* < 0.01].

**Table 6:** Result of the LME model analysis for reaction times.

Variables	Estimate	SE	df	*t* value	Pr(>|t|)	*p*-value
(Intercept)	0.35	0.03	95.52	12.96	*p* < 0.001	***
VO_ABS_ S_ERG_	0.12	0.02	1127.06	5.53	*p* < 0.001	***
S_TOP_VO_ABS_	0.06	0.02	1122.14	2.77	*p* < 0.01	**
O_TOP_VS_ERG_	0.04	0.02	1131.53	1.93	*p* = 0.054	
trial.z	−0.01	0.02	50.06	−0.85	*p* = 0.401	

Participants = 40. Item = 32. Total Observation = 1,218. lmer(log(rt) ~ wordorder + trial.z + (0 + trial.z|participant) + (1|participant) + (0 + trial.z|participant) + (1|item), data).

**Figure 7:**
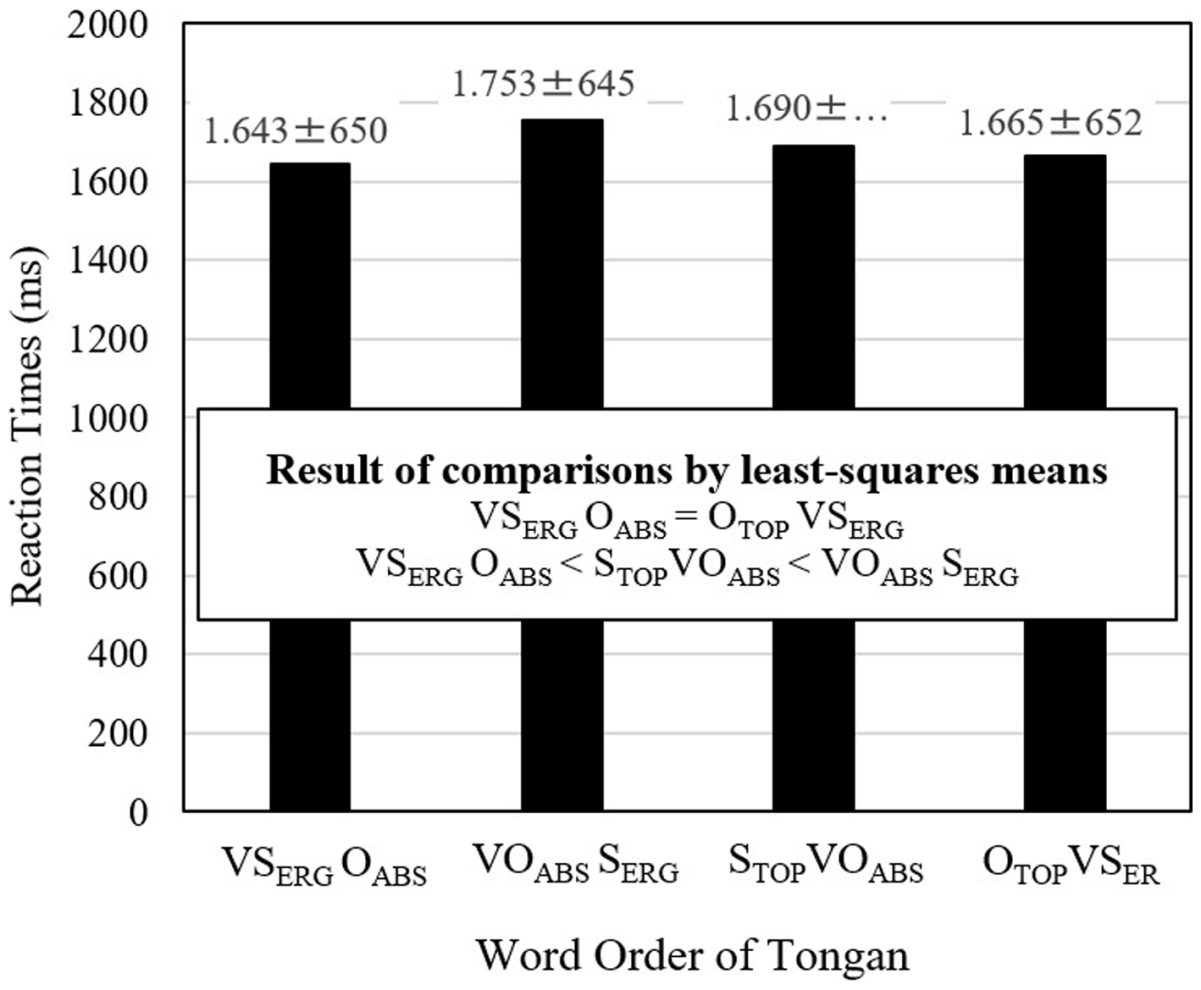
Reaction times of the four word orders of Tongan sentences. The values after ± refer to standard errors.

To examine differences among word orders more in-depth, reaction times for the four word orders were compared using the *R* package *lsmeans* (Searle et al., 1980). The means and standard deviations are reported, and the results of multiple comparisons are shown in Figure 7. The result indicated that O_TOP_VS_ERG_ object topicalized sentences (M = 1,665 ms) were processed equally as quickly as VS_ERG_O_ABS_ canonical sentences (M = 1,643 ms). However, S_TOP_VO_ABS_ subject topicalized sentences (M = 1,690 ms) were processed significantly more slowly than VS_ERG_O_ABS_ canonical sentences. Furthermore, S_TOP_VO_ABS_ sentences were processed significantly faster than VO_ABS_S_ERG_ scrambled sentences (M = 1,753 ms). Thus, scrambled sentences were processed more slowly than both object and subject topicalized sentences.

### 4.2 Discussion

Experiment 3 addressed the primary objective of this study: examining the effects of scrambling and topicalization in Tongan sentences. The efficiency of processing for the four word orders (VSO, VOS, SVO, and OVS) was measured in terms of speed and accuracy. Similar to Experiment 2, the results indicated that VSO canonical sentences were processed more rapidly and accurately than VOS scrambled sentences, supporting the existence of a scrambling effect. Interestingly, OVS object topicalized sentences were processed as swiftly and accurately as VSO canonical sentences, while SVO subject topicalized sentences were processed more slowly than VSO canonical sentences but faster than VOS scrambled sentences. Both SVO subject and OVS object topicalized sentences were processed more efficiently than VOS scrambled sentences. The object topicalization sentences exhibited a positive (efficient) processing effect, whereas the subject topicalization sentences were processed less efficiently than object topicalization sentences but more efficiently than VOS scrambled sentences. These findings did not entirely align with Prediction 2 based on the filler-gap dependency. Further details were discussed in the subsequent General Discussion section.

## 5 General discussion

The present study conducted three experiments to investigate differences in the effects of canonical/scrambled and subject/object topicalization on the verb-final language of Korean and the verb-initial language of Tongan. Experiment 1 focused on Korean to compare the findings with those of Imamura et al. (2016) on Japanese. Experiment 1 supported Prediction 1 with the exception of no difference observed between O_ACC_ S_NOM_V and O_TOP_ S_NOM_V. Consistent with the findings of the previous Japanese experiment (Imamura et al., 2016), the OSV scrambled order in Korean was processed more slowly than the SOV canonical order (i.e., scrambling effect). The SVO subject topicalized order was processed at a comparable speed to the SOV canonical order, suggesting the SOV subject topicalized order in Korean might have processing such as the canonical sentence. However, unlike Prediction 1, there was no noticeable difference in processing time between object topicalized and scrambled sentences in Korean. This discrepancy between Japanese and Korean could be attributed to the animacy effect.

Given that the SOV canonical order in Korean overlapped with the subject topicalized order, and the OSV scrambled order in Korean overlapped with the object topicalized order, Tongan exhibits a distinct word order for canonical/scrambled and subject/object topicalization. Consequently, the study proceeded to investigate the head-initial language of Tongan. Experiment 2 employed a phrase-by-phrase processing experiment to determine whether the VSO order is canonical in Tongan. The results of Experiment 2 indicated that native Tongan speakers process the VSO order faster than the VOS order, confirming the canonical status of the VSO order in Tongan (scrambling effect, V S_ERG_ O_ABS_ < V O_ABS1_ S_ERG_ *gap*_1_), which aligns with previous linguistic studies (Churchward, 1953; Dixon, 1979, 1994; Otsuka, 2000, 2005a,b, 2010). Experiment 2 provided support for the scrambling effects outlined in Prediction 2.

The primary focus of the present study was Experiment 3, which investigated the effect of subject/object topicalization separately from the canonical/scrambled effect. Processing efficiency, in terms of both speed and accuracy, was assessed for the four word orders (VSO, VOS, SVO, and OVS). The findings of Experiment 3, outlined below, shed light on Tongan sentence processing:

**Results of experiment 3: Tongan sentence processing:**

$$\begin{array}{l} V\;S_{\mathrm{ERG}}\;O_{\mathrm{ABS}}=\\ O_{\mathrm{TOP}1}\;V\;S_{\mathrm{ERG}}\;gap_{1}<S_{\mathrm{TOP}1}\;V\;gap_{1}\;O_{\mathrm{ABS}}<V\;O_{\mathrm{ABS}1}\;S_{\mathrm{ERG}}\;gap_{1}\text{.} \end{array}$$

The results of Experiment 3 did not entirely align with Prediction 2. A notable discrepancy was observed: OVS object topicalized sentences were unexpectedly processed as swiftly and accurately as VSO canonical sentences. Additionally, VSO canonical sentences were processed more rapidly and accurately than VOS scrambled sentences, whereas SVO subject topicalized sentences were processed more slowly than VSO canonical sentences but faster than VOS scrambled sentences. Both SVO subject and OVS object topicalized sentences were processed more efficiently than VOS scrambled sentences. Consequently, Prediction 2, which was based on syntactic complexity according to the filler-gap dependency, or in other words, the movement-based anticipation, was not supported. Further detailed discussion will be provided in the following sections.

### 5.1 Korean sentence processing in comparison to Japanese

Various syntactic similarities are found between Korean and Japanese. In both languages, a scrambled order is created by moving the object in front of the subject, as in O_1_SV*gap*_1_. The result of Experiment 1 indicated that the scrambled O_1_SV*gap*_1_ order in Korean was processed more slowly and less accurately than the SOV canonical order. This result confirmed the existence of the scrambling effect, as also found in previous studies on Japanese sentence processing (e.g., Mazuka et al., 2002; Ueno and Kluender, 2003; Koizumi and Tamaoka, 2004, 2010; Miyamoto and Takahashi, 2004; Tamaoka et al., 2005, 2014; Imamura et al., 2016; Witzel and Witzel, 2016; Tamaoka and Mansbridge, 2019). The processing delay for the syntactic structure of the O_1_SV*gap*_1_ scrambled order in both Japanese and Korean could be explained by the *gap-filling parsing* model (Frazier and Rayner, 1982; Stowe, 1986; Frazier, 1987; Frazier and Clifton, 1989; Frazier and Flores D’Arcais, 1989) which was presented in the introductory section of this article.

Regarding topicalization, in agreement with Imamura et al. (2016), the subject topicalized order in Korean was processed equally as quickly as the canonical order. However, it is still uncertain whether the processing speed of subject topicalization is accelerated by the influence of the SOV canonical order. Additionally, there were no differences in processing speed between the object topicalized order and the scrambled order in Korean. Once again, since the OSV scrambled order overlaps with the object topicalized order, the null difference result may have been caused by the influence of the OSV scrambled order and bears no relation to the object topicalization effect. It is quite possible that the scrambling effect may override the topicalization effect. It is also conceivable that the particle -*eun/neun* can be a pseudo-subject. This might be a partial reason for the fact that no difference has been found in this study between S_NOM_OV and S_TOP_OV in accuracy and reaction time.

One difference was found between Japanese and Korean. There was no difference in processing speed between the object topicalized and the scrambled order in Korean. By contrast, the object topicalized order was slower than the scrambled order in Japanese. This different result may be created by the animacy effect (Mak et al., 2002; Hsiao and Gibson, 2003; Pu, 2007; Vasishth et al., 2013; Kwon et al., 2019). In the Japanese study (Imamura et al., 2016), proper nouns such as *Satoo* and *Suzuki* were used for both subjects and objects. For example, サトウがスズキを褒めた (*Satoo-ga Suzuki-o home-ta*) meaning ‘Sato praised Suzuki’ contains two animate nouns. These two animate proper nouns can function as either subject or object. Unlike the Japanese study, the stimulus sentences used in the current Experiment 1 using Korean were mostly constructed by pairing an animate noun with an inanimate noun, such as in 여성이 물을 삼켰다 (*Yeoseong-i mul-ul sam-keossda*) meaning ‘The woman drank water.’ This animacy contrast between the subject and the object in Korean may have facilitated the processing of OSV object topicalized order, which is a plausible explanation for why there was no difference in the speed of sentence processing between OVS object topicalized and OSV scrambled orders. However, the animacy of noun phrases could be closely related to thematic assignments and argument structures (Comrie, 1981; Mak et al., 2002; Traxler et al., 2002, 2005; Bornkessel-Schlesewsky and Schlesewsky, 2006; Gennari and MacDonald, 2008; Wagers and Phillips, 2014; Ness and Meltzer-Asscher, 2017). This issue should be investigated in future studies.

### 5.2 The scrambling effect in Tongan

Previous studies (e.g., Churchward, 1953; Dixon, 1979, 1994; Otsuka, 2000, 2005a,b, 2010; Custis, 2004) have suggested that the canonical order in Tongan is VSO, while the scrambled order is VOS. However, no large-scale corpus study has been conducted in Tongan to verify that the VSO order is dominant. Thus, before investigating the processing of subject/object topicalized sentences, a maze task was conducted in Experiment 2 to determine whether VSO is truly the canonical order of Tongan transitive sentences. The lexical maze task is a unique task in which participants make a series of continuous lexical decisions that reveal how they are processing sentences without the participants’ awareness of this background operation. This task makes it possible to compare phrase-by-phrase sentence processing between VSO and VOS orders.

Because the first phrase consisting of the adverb *mahalo* (‘maybe’) and the second phrase containing a verb is the same in *Adv*VSO canonical and *Adv*VOS scrambled sentences in the lexical maze task, no differences in processing speed for the first and second phrases were expected. The phrases of interest appear after the verb. In the third subject/object phrase, the noun with the ergative ‘*e* marker (S) was processed faster than the noun with the absolutive ‘*a* marker (O). Since VSO is considered the most used order, the subject would be expected to appear after the verb. In the following fourth object/subject phrase, the noun with the absolutive ‘*a* marker (O) was processed faster than the noun with the ergative ‘*e* marker (S). This result was surprising because after the third phrase was identified, the following final fourth phrase would automatically have been an object for the canonical or a subject for the scrambled order. Even after the processing of the third phrase, scrambling continues to affect the processing of the sentence-final subject. This could be due to the search for the *gap* after the subject in the *Adv*VO_1_S*gap*_1_. As anticipated by Prediction 2, the processing difference between VSO and VOS in the third and fourth phrases in Experiment 2 supports the proposal that VSO is the canonical order.

The effect of scrambling in Experiment 2 was further confirmed in Experiment 3. The VSO canonical order was processed more quickly and accurately than the VOS scrambled order. As Otsuka (2005b, 2010) proposed, VOS scrambled order in Tongan is understood as movement (VO_1_S*gap*_1_) motivated by a new information focus. Otsuka argues that the position immediately following the verb is reserved for new information. In other words, given a context in which the object is new information and the subject is old information (e.g., answering an object *wh*-question), the object is placed before the subject. According to this proposal, as in Japanese and Korean, gap-filling parsing (Stowe, 1986; Frazier, 1987; Frazier and Clifton, 1989; Frazier and Flores D’Arcais, 1989; Traxler and Pickering, 1996) may have been used for processing the Tongan VOS scrambled order. Consequently, Experiments 2 and 3 provided clear evidence for the linguistic claim that the VSO order is canonical and the VOS is scrambled in Tongan (Churchward, 1953; Dixon, 1979, 1994; Otsuka, 2000, 2005a,b, 2010).

Furthermore, some Tongan verbs can be used both as transitive and intransitive verbs (i.e., ambitransitivity). Since Tongan is an ergative language and the absolutive case marker ‘*a* also marks the subject of intransitive sentences, the VO_ABS_ order can be interpreted as being an intransitive canonical sentence in VS_ABS_ order. When a noun phrase marked by the ergative case *e*’ follows, the sentence is considered a transitive VO_ABS_S_ERG_ scrambled sentence. In that case, it may be that the delay in processing a VO_ABS_S_ERG_ ordered sentence is caused by verb transitivity confusion. Nine of 32 verbs in Experiment 3 fall into this ambitransitivity category; these are *kai* ‘eat’, *talitali* ‘wait’, *ui* ‘call’, *tuli* ‘chase’, *huo* ‘hoe’, *fo* ‘wash clothes’, *inu* ‘drink’, *tui* ‘wear’, and *lau* ‘read.’ However, the nouns used in Experiment 3 are basically a pair of animate nouns for the subject (e.g., ‘woman’, ‘teacher’, ‘children’) and inanimate nouns (e.g., ‘clothes’, ‘bicycle’, ‘money’) for the object. Transitivity confusion could be avoided by using animacy contrast in the stimulus sentences in Experiment 3. Thus, we do not consider the possibility of the influence of ambitransitivity to be very likely. Hence, it would be parsimonious to conclude that the differences in accuracy and speed were primarily driven by the effect of scrambling order: specifically, VOS scrambled order exhibited a 4.07% lower accuracy rate and was 110 ms slower in speed compared to the VSO canonical order.

### 5.3 Processing SVO/OVS topicalized sentences in Tongan

The SVO and OVS sentence-fronting topicalized orders are always *ko*-marked (Custis, 2004), as shown in Sentences (7) and (8) above. Custis (2004) considers that both SVO and OVS orders involve A-bar movement similar to relativization. If that is the case, then both orders would be predicted to be recognized by native Tongan speakers as a more complex syntactic structure than the VSO canonical order.

According to the gap-filling parsing model (Stowe, 1986; Frazier, 1987; Frazier and Clifton, 1989; Frazier and Flores D’Arcais, 1989; Traxler and Pickering, 1996), the processing of Tongan topicalized sentences is described as follows: a topicalized subject phrase is moved in front of the verb; Tongan speakers recognize the initial topicalized phrase as the filler, and then look for its original position in the specifier of *gap* to establish the filler-gap dependency. In this framework, the OVS order should have a longer processing time than the SVO order because the movement involved in the object topicalization O_1_VS*gap*_1_ is greater in distance than the movement involved in the subject topicalization S_1_V*gap*_1_O. In addition, the object relative clause (O_1_VS*gap*_1_ in Tongan) for many of the world languages has been found to be more difficult to process and comprehend than the subject-extracted (S_1_V*gap*_1_ in Tongan) relative clause (e.g., Staub, 2010; Jäger et al., 2015).

However, an unexpected result was found in Experiment 3. There was no significant difference in processing speed and accuracy between the OVS object tropicalized order and the VSO canonical order (OVS=VSO). By contrast, the processing of the SVO subject topicalized order was slower than for the VSO canonical order (VSO < SVO) but faster than for the VOS scrambled order (SVO < OVS). The contrasting result between SVO and OVS may be related to the prominence of the topic. Custis (2004) considers Tongan to be a topic-marking language. A topic can be placed in one of two positions in Tongan—either immediately before the verb (SVO/OVS) or immediately after the verb (VOS). The placement of a topic is pragmatically motivated and syntactically results in one of the three-word orders of SVO, OVS, or VOS, while the VSO canonical order is pragmatically neutral. Custis (2004) observed (using the corpus data) that SVO and OVS orders are used to mark less salient topics and VOS to mark more salient topics in discourse. By contrast, the VOS scrambled order is used for presenting a salient topic. The activation levels infer cognitive status reflecting the frequency of a discourse topic: namely, how often would it be talked about? When the NP is frequently mentioned in discourse, it is more accessible and hence less salient in the discourse. This is the case for the fronted subject of SVO or the fronted object of OVS orders. On the other hand, a low activation level indicates that the topic is less frequently talked about so that it would be more salient and less accessible. This is the case for the object of VOS scrambled order.

The idea of cognitive status by Custis (2004) may account for the result of the processing speed in VSO < SVO < VOS. The pragmatically-neutral VSO is the fastest, while the VOS scrambled order is the slowest. The SVO subject topicalized order falls in between the VSO and VOS orders. Then, what causes the difference in processing speed between SVO and OVS orders? One possibility is that, as shown in Sentence (7), the resumptive pronoun *ne* appears after the tense and before the verb in the case of subject topicalization, so this visible syntactic feature may require extra processing time than the OVS object topicalized order with no resumptive pronoun. Prediction 2 (i.e., VSO < VOS = SVO < OVS) was constructed according to the syntactic complexity constructed by the filler-gap dependency (Stowe, 1986; Frazier, 1987; Frazier and Clifton, 1989; Frazier and Flores D’Arcais, 1989; Traxler and Pickering, 1996), regarding the word order in Tongan scrambling and topicalization. However, the results of Experiment 3 (i.e., VSO = OVS < SVO < VOS) indicated that word orders of both subject and object topicalization facilitated in sentence processing, deviating from the movement-based anticipation of Prediction 2.

In conclusion, the present study sheds light on the processing dynamics of Tongan sentence structures, offering insights into the cognitive mechanisms underlying language comprehension. The observed processing speeds, with VSO being the fastest, followed by SVO and then VOS, suggest a nuanced interplay between syntactic structure and cognitive processing. The unexpected processing efficiency of OVS orders compared to the predicted pattern raises intriguing questions about the underlying mechanisms at play. The discrepancy between the observed results and Prediction 2 highlights the limitations of relying solely on syntactic complexity metrics such as the filler-gap dependency. Instead, the findings suggest that the facilitation effect of both subject and object topicalization in Tongan may be better explained by a broader consideration of cognitive status and pragmatic factors. Further investigations into the processing dynamics of topicalized word orders in Tongan, as well as comparative studies across different languages, are warranted to deepen our understanding of how linguistic structure interacts with cognitive processing mechanisms. This study opens avenues for future research into the intricate interplay between syntax, cognition, and language comprehension.

## Data availability statement

The raw data supporting the conclusions of this article will be made available by the authors, without undue reservation.

## Ethics statement

The studies involving humans were approved by Research Ethics Committee of Busan National University (Exp 1) Research Ethics Committee of Tohoku University (Exp 2 and 3). The studies were conducted in accordance with the local legislation and institutional requirements. The participants provided their written informed consent to participate in this study.

## Author contributions

KT: Conceptualization, Data curation, Formal analysis, Investigation, Methodology, Project administration, Resources, Software, Supervision, Validation, Visualization, Writing – original draft, Writing – review & editing. SY: Data curation, Formal analysis, Methodology, Writing – original draft, Writing – review & editing. JZ: Data curation, Writing – original draft, Writing – review & editing. YO: Conceptualization, Data curation, Investigation, Writing – original draft, Writing – review & editing. HL: Data curation, Writing – original draft, Writing – review & editing. MK: Funding acquisition, Resources, Writing – original draft, Writing – review & editing. RV: Resources, Supervision, Validation, Writing – original draft, Writing – review & editing.

## Footnotes

^1^https://pavlovia.org/
