# Supplementary material for: Syntactic structures in motion: investigating word order variations in verb-final (Korean) and verb-initial (Tongan) languages
Source: Front Psychol. 2024 Apr 24;15:1360191. doi: 10.3389/fpsyg.2024.1360191 (PMC11078219; doi:10.3389/fpsyg.2024.1360191)
Supplement: Supplementary file 1 [file Table_1.DOCX]

Supplementary Material

**Appendix 1 – Krean Stimulus Sentences Used for the Sentence Correctness Decision Task in Experiment 1**

All 32 pairs of correct sentences containing three phrases are listed below. In the following sentence gloss, V refers to a verb, NP refers to a noun phrase, PST refers to the past tense, NOM refers to a nominative case marker, ACC refers to an accusative case maker, and TOP refers to a topicalization maker.

(1a) SOV canonical order

여성이 물을 삼켰다.

Yeo-seong-i mul-eul sam-kyeoss-da.

S(woman)-NOM NP(water)-ACC NP(drink)-PST

‘The woman drank water.’

(1b) OSV scrambled order

물을 여성이 삼켰다.

Mul-eul yeo-seong-i sam-kyeoss-da.

NP(water)-ACC S(woman)-NOM NP(drink)-PST

(1c) SOV subject topicalization

여성은 물을 삼켰다.

Yeo-seong-eun mul-eul sam-kyeoss-da.

S(woman)-TOP NP(water)-ACC NP(drink)-PST

(1d) OSV object topicalization

물은 여성이 삼켰다.

Mul-eun yeo-seong-i sam-kyeoss-da.

NP(water)-TOP S(woman)-NOM NP(drink)-PST

Hereafter, only Korean sentence pairs and their translations are listed below.

(2a) 경찰이 범인을 잡았다. ‘The police caught the criminal.’

　　Gyeong-chal-i beom-in-eul jab-ass-da.

(2b) 범인을 경찰이 잡았다.

Beom-in-eul gyeong-chal-i jab-ass-da.

(2c) 경찰은 범인을 잡았다.

Gyeong-chal-eun beom-in-eul jab-ass-da.

(2d) 범인은 경찰이 잡았다.

　　Beom-in-eun gyeong-chal-i jab-ass-da.

(3a) 할머니가 전화를 걸었다. ‘The grandma made a telephone call.’

　　Hal-meo-ni-ga jeon-hwa-leul geol-eoss-da.

(3b) 전화를 할머니가 걸었다.

　　 Jeon-hwa-leul hal-meo-ni-ga geol-eoss-da.

(3c) 할머니는 전화를 걸었다.

Hal-meo-ni-neun jeon-hwa-leul geol-eoss-da.

(3d) 전화는 할머니가 걸었다.

　　 Jeon-hwa-neun hal-meo-ni-ga geol-eoss-da.

(4a) 미용사가 의자를 옮겼다. ‘The hairdresser carried the chair.

Mi-yong-sa-ga ui-ja-leul olm-gyeoss-da.

(4b) 의자를 미용사가 옮겼다.

Ui-ja-leul mi-yong-sa-ga olm-gyeoss-da.

(4c) 미용사는 의자를 옮겼다.

　　Mi-yong-sa-neun ui-ja-leul olm-gyeoss-da.

(4d) 의자는 미용사가 옮겼다.

　　Ui-ja-neun mi-yong-sa-ga olm-gyeoss-da.

(5a) 선수가 볼을 쳤다. ‘The player hit the ball.’

Seon-su-ga bol-eul chyeoss-da.

(5b) 볼을 선수가 쳤다.

　　Bol-eul seon-su-ga chyeoss-da.

(5c) 선수는 볼을 쳤다.

　　Seon-su-neun bol-eul chyeoss-da.

(5d) 볼은 선수가 쳤다.

　　Bol-eun seon-su-ga chyeoss-da.

(6a) 남자가 옷을 빨았다. ‘The man washed his clothes.’

　　Nam-ja-ga os-eul ppal-ass-da.

(6b) 옷을 남자가 빨았다.

Os-eul nam-ja-ga ppal-ass-da.

(6c) 남자는 옷을 빨았다.

　　Nam-ja-neun os-eul ppal-ass-da.

(6d) 옷은 남자가 빨았다.

　　Os-eun nam-ja-ga ppal-ass-da.

(7a) 어머니가 쓰레기를 치웠다. ‘The mother threw away the trash.’

Eo-meo-ni-ga sseu-le-gi-leul chi-woss-da.

(7b) 쓰레기를 어머니가 치웠다.

Sseu-le-gi-leul eo-meo-ni-ga chi-woss-da.

(7c) 어머니는 쓰레기를 치웠다.

Eo-meo-ni-neun sseu-le-gi-leul chi-woss-da.

(7d) 쓰레기는 어머니가 치웠다.

Sseu-le-gi-neun eo-meo-ni-ga chi-woss-da.

(8a) 삼촌이 모자를 벗었다. ‘The uncle took off his hat.’

Sam-chon-i mo-ja-leul beos-eoss-da.

(8b) 모자를 삼촌이 벗었다.

Mo-ja-leul sam-chon-i beos-eoss-da.

(8c) 삼촌은 모자를 벗었다.

　　Sam-chon-eun mo-ja-leul beos-eoss-da.

(8d) 모자는 삼촌이 벗었다.

　　Mo-ja-neun sam-chon-i beos-eoss-da.

(9a) 아버지가 고기를 잘랐다. ‘The father cut the meat.’

　　A-beo-ji-ga go-gi-leul jal-lass-da.

(9b) 고기를 아버지가 잘랐다.

　　 Go-gi-leul a-beo-ji-ga jal-lass-da.

(9c) 아버지는 고기를 잘랐다.

　　 A-beo-ji-neun go-gi-leul jal-lass-da.

(9d) 고기는 아버지가 잘랐다.

　　 Go-gi-neun a-beo-ji-ga jal-lass-da.

(10a) 언니가 파이를 구웠다. ‘The sister baked a pie.’

Eon-ni-ga pa-i-leul gu-woss-da.

(10b) 파이를 언니가 구웠다.

Pa-i-leul eon-ni-ga gu-woss-da.

(10c) 언니는 파이를 구웠다.

Eon-ni-neun pa-i-leul gu-woss-da.

(10d) 파이는 언니가 구웠다.

Pa-i-neun eon-ni-ga gu-woss-da.

(11a) 운전수가 창문을 열었다. ‘The driver opened the window.’

Un-jeon-su-ga chang-mun-eul yeol-eoss-da.

(11b) 창문을 운전수가 열었다.

Chang-mun-eul un-jeon-su-ga yeol-eoss-da.

(11c) 운전수는 창문을 열었다.

Un-jeon-su-neun chang-mun-eul yeol-eoss-da.

(11d) 창문은 운전수가 열었다.

Chang-mun-eun un-jeon-su-ga yeol-eoss-da.

(12a) 학생이 책을 읽었다. ‘The student read a book.’

Hag-saeng-i chaeg-eul ilg-eoss-da.

(12b) 책을 학생이 읽었다.

Chaeg-eul hag-saeng-i ilg-eoss-da.

(12c) 학생은 책을 읽었다.

Hag-saeng-eun chaeg-eul ilg-eoss-da.

(12d) 책은 학생이 읽었다.

Chaeg-eun hag-saeng-i ilg-eoss-da.

(13a) 기술자가 에어콘을 수리했다. ‘The technician repaired the air conditioner.’

Gi-sul-ja-ga e-eo-kon-eul su-li-haess-da

(13b) 에어콘을 기술자가 수리했다.

E-eo-kon-eul gi-sul-ja-ga su-li-haess-da.

(13c) 기술자는 에어콘을 수리했다.

Gi-sul-ja-neun e-eo-kon-eul su-li-haess-da.

(13d) 에어콘은 기술자가 수리했다.

E-eo-kon-eun gi-sul-ja-ga su-li-haess-da.

(14a) 화가가 꽃을 그렸다. ‘The painter painted flowers.’

Hwa-ga-ga kkoch-eul geu-lyeoss-da.

(14b) 꽃을 화가가 그렸다.

Kkoch-eul hwa-ga-ga geu-lyeoss-da.

(14c) 화가는 꽃을 그렸다.

Hwa-ga-neun kkoch-eul geu-lyeoss-da.

(14d) 꽃은 화가가 그렸다.

Kkoch-eun hwa-ga-ga geu-lyeoss-da.

(15a) 신부가 촛불을 불었다. ‘The bride blew out the candles.’

Sin-bu-ga chos-bul-eul bul-eoss-da.

(15b) 촛불을 신부가 불었다.

Chos-bul-eul sin-bu-ga bul-eoss-da.

(15c) 신부는 촛불을 불었다.

Sin-bu-neun chos-bul-eul bul-eoss-da.

(15d) 촛불은 신부가 불었다.

Chos-bul-eun sin-bu-ga bul-eoss-da.

(16a) 오빠가 잔디를 깎았다. ‘The brother mowed the lawn.’

O-ppa-ga jan-di-leul kkakk-ass-da.

(16b) 잔디를 오빠가 깎았다.

Jan-di-leul o-ppa-ga kkakk-ass-da.

(16c) 오빠는 잔디를 깎았다.

O-ppa-neun jan-di-leul kkakk-ass-da.

(16d) 잔디는 오빠가 깎았다.

Jan-di-neun o-ppa-ga kkakk-ass-da.

(17a) 목수가 집을 지었다. ‘The carpenter built the house.’

Mog-su-ga jib-eul ji-eoss-da.

(17b) 집을 목수가 지었다.

Jib-eul mog-su-ga ji-eoss-da.

(17c) 목수는 집을 지었다.

Mog-su-neun jib-eul ji-eoss-da.

(17d) 집은 목수가 지었다.

Jib-eun mog-su-ga ji-eoss-da.

(18a) 아기가 우유를 마셨다. ‘The baby drank milk.’

A-gi-ga u-yu-leul ma-syeoss-da.

(18b) 우유를 아기가 마셨다.

U-yu-leul a-gi-ga ma-syeoss-da.

(18c) 아기는 우유를 마셨다.

A-gi-neun u-yu-leul ma-syeoss-da.

(18d) 우유는 아기가 마셨다.

U-yu-neun a-gi-ga ma-syeoss-da.

(19a) 군인이 신발을 신었다. ‘The soldier put on (his) shoes.’

Gun-in-i sin-bal-eul sin-eoss-da.

(19b) 신발을 군인이 신었다.

Sin-bal-eul gun-in-i sin-eoss-da.

(19c) 군인은 신발을 신었다.

Gun-in-eun sin-bal-eul sin-eoss-da.

(19d) 신발은 군인이 신었다.

Sin-bal-eun gun-in-i sin-eoss-da.

(20a) 조카가 불을 붙였다. ‘(My) nephew lit the fire.’

Jo-ka-ga bul-eul but-yeoss-da.

(20b) 불을 조카가 붙였다.

Bul-eul jo-ka-ga but-yeoss-da.

(20c) 조카는 불을 붙였다.

Jo-ka-neun bul-eul but-yeoss-da.

(20d) 불은 조카가 붙였다.

Bul-eun jo-ka-ga but-yeoss-da.

(21a) 남동생이 연필을 깎았다. ‘(My) brother sharpened his pencil.’

Nam-dong-saeng-i yeon-pil-eul kkakk-ass-da.

(21b) 연필을 남동생이 깎았다.

Yeon-pil-eul nam-dong-saeng-i kkakk-ass-da.

(21c) 남동생은 연필을 깎았다.

Nam-dong-saeng-eun yeon-pil-eul kkakk-ass-da.

(21d) 연필은 남동생이 깎았다.

Yeon-pil-eun nam-dong-saeng-i kkakk-ass-da.

(22a) 아줌마가 요리를 날랐다. ‘(My) aunt brought the dishes.’

A-jum-ma-ga yo-li-leul nal-lass-da.

(22b) 요리를 아줌마가 날랐다.

Yo-li-leul a-jum-ma-ga nal-lass-da.

(22c) 아줌마는 요리를 날랐다.

A-jum-ma-neun yo-li-leul nal-lass-da.

(22d) 요리는 아줌마가 날랐다.

Yo-li-neun a-jum-ma-ga nal-lass-da.

(23a) 소방수가 불을 껐다. ‘The fireman put out the fire.’

So-bang-su-ga bul-eul kkeoss-da.

(23b) 불을 소방수가 껐다.

Bul-eul so-bang-su-ga kkeoss-da.

(23c) 소방수는 불을 껐다.

So-bang-su-neun bul-eul kkeoss-da.

(23d) 불은 소방수가 껐다.

Bul-eun so-bang-su-ga kkeoss-da.

(24a) 의사가 주사를 놓았다. ‘The doctor gave an injection.’

Ui-sa-ga ju-sa-leul noh-ass-da.

(24b) 주사를 의사가 놓았다.

Ju-sa-leul ui-sa-ga noh-ass-da.

(24c) 의사는 주사를 놓았다.

Ui-sa-neun ju-sa-leul noh-ass-da.

(24d) 주사는 의사가 놓았다.

Ju-sa-neun ui-sa-ga noh-ass-da.

(25a) 학생이 교실을 나갔다. ‘The student left the classroom.’

Hag-saeng-i gyo-sil-eul na-gass-da.

(25b) 교실을 학생이 나갔다.

Gyo-sil-eul hag-saeng-i na-gass-da.

(25c) 학생은 교실을 나갔다.

Hag-saeng-eun gyo-sil-eul na-gass-da.

(25d) 교실은 학생이 나갔다.

Gyo-sil-eun hag-saeng-i na-gass-da.

(26a) 친구가 편지를 썼다. ‘(My) friend wrote a letter.’

Chin-gu-ga pyeon-ji-leul sseoss-da.

(26b) 편지를 친구가 썼다.

Pyeon-ji-leul chin-gu-ga sseoss-da.

(26c) 친구는 편지를 썼다.

Chin-gu-neun pyeon-ji-leul sseoss-da.

(26d) 편지는 친구가 썼다.

Pyeon-ji-neun chin-gu-ga sseoss-da.

(27a) 약사가 약을 버렸다. ‘The pharmacist threw the medicine away.’

Yag-sa-ga yag-eul beo-lyeoss-da.

(27b) 약을 약사가 버렸다.

Yag-eul yag-sa-ga beo-lyeoss-da.

(27c) 약사는 약을 버렸다.

Yag-sa-neun yag-eul beo-lyeoss-da.

(27d) 약은 약사가 버렸다.

Yag-eun yag-sa-ga beo-lyeoss-da.

(28a) 소년이 자전거를 훔쳤다. ‘The boy stole the bike.’

So-nyeon-i ja-jeon-geo-leul hum-chyeoss-da.

(28b) 자전거를 소년이 훔쳤다.

Ja-jeon-geo-leul so-nyeon-i hum-chyeoss-da.

(28c) 소년은 자전거를 훔쳤다.

So-nyeon-eun ja-jeon-geo-leul hum-chyeoss-da.

(28d) 자전거는 소년이 훔쳤다.

Ja-jeon-geo-neun so-nyeon-i hum-chyeoss-da.

(29a) 여동생이 가방을 숨겼다. ‘(My) sister hid her bag.’

Yeo-dong-saeng-i ga-bang-eul sum-gyeoss-da.

(29b) 가방을 여동생이 숨겼다.

Ga-bang-eul yeo-dong-saeng-i sum-gyeoss-da.

(29c) 여동생은 가방을 숨겼다.

Yeo-dong-saeng-eun ga-bang-eul sum-gyeoss-da.

(29d) 가방은 여동생이 숨겼다.

Ga-bang-eun yeo-dong-saeng-i sum-gyeoss-da.

(30a) 남편이 총을 발견했다. ‘(My) husband found the gun.’

Nam-pyeon-i chong-eul bal-gyeon-haess-da.

(30b) 총을 남편이 발견했다.

Chong-eul nam-pyeon-i bal-gyeon-haess-da.

(30c) 남편은 총을 발견했다.

Nam-pyeon-eun chong-eul bal-gyeon-haess-da.

(30d) 총은 남편이 발견했다.

Chong-eun nam-pyeon-i bal-gyeon-haess-da.

(31a) 아내가 케이크를 만들었다. ‘(My) wife made the cake.’

A-nae-ga ke-i-keu-leul man-deul-eoss-da.

(31b) 케이크를 아내가 만들었다.

　　 Ke-i-keu-leul a-nae-ga man-deul-eoss-da.

(31c) 아내는 케이크를 만들었다.

　　  A-nae-neun ke-i-keu-leul man-deul-eoss-da.

(31d) 케이크는 아내가 만들었다.

Ke-i-keu-neun a-nae-ga man-deul-eoss-da.

(32a) 남자가 밧줄을 당겼다. ‘The man pulled the rope.’

Nam-ja-ga bas-jul-eul dang-gyeoss-da.

(32b) 밧줄을 남자가 당겼다.

Bas-jul-eul nam-ja-ga dang-gyeoss-da.

(32c) 남자는 밧줄을 당겼다.

Nam-ja-neun bas-jul-eul dang-gyeoss-da.

(32d) 밧줄은 남자가 당겼다.

Bas-jul-eun nam-ja-ga dang-gyeoss-da.

**Appendix 2 – Tongan Stimulus Sentences Used for the Maze Task in Experiment 2**

All 30 pairs of correct sentences containing four phrases including an initially presented adverb (Adv) *mahalo* ‘maybe’ are listed below. In the following sentence gloss, V refers to a verb, NP refers to a noun phrase, PST refers to the past tense, ABS (*'a*) refers to an absolute case marker, ERG (*'e*) refers to an absolute ergative case maker.

(1a) AdvVSO canonical order

*Mahalo na'e taa'i 'e Sione 'a Lu'isa.*

Adv(maybe) V(hit)-PAST NP-ERG (Sione) NP-ABS (Lu'isa)

‘Maybe Sione hit Luisa.’

(1b) AdvVOS scrambled order

*Mahalo na'e taa'i 'a Sione 'e Lu'isa.*

Adv(maybe) V(hit)-PAST NP-ABS (Sione) NP-ERG (Lu'isa)

‘Maybe Luisa hit Sione.’

Hereafter, only Tongan sentence pairs and their translations of (a) and (b) without the initially-presented adverb *mahalo* ‘maybe’ task are listed.

(2a) Na'e 'akahi 'e Fifita 'a Pele. ‘Fifita kicked Pele.’

(2b) Na'e 'akahi 'a Fifita 'e Pele. ‘Pele kicked Fifita.’

(3a) Na'e tamate'i 'e Uili 'a Sina. ‘Uili killed Sina.’

(3b) Na'e tamate'i 'a Uili 'e Sina. ‘Sina killed Uili.’

(4a) Na'e tui'i 'e Fili 'a Ma'ake. ‘Fili ran over Ma’ake.’

(4b) Na'e tui'i 'a Fili 'e Ma'ake. ‘Ma’ake ran over Fili.’

(5a) Na'e talitali 'e Kaufusi 'a Taniela. ‘Kaufusi welcomed Taniela.’

(5b) Na'e talitali 'a Kaufusi 'e Taniela. ‘Taniela welcomed Kaufusi.’

(6a) Na'e lohiaki'i 'e Make 'a Fane. ‘Make lied to Fane.’

(6a) Na'e lohiaki'i 'a Make 'e Fane. ‘Fane lied to Make.’

(7a) Na'e teke'i 'e Samuela 'a Sefita. ‘Samuela pushed Sefita.’

(7b) Na'e teke'i 'a Samuela 'e Sefita. ‘Sefita pushed Samuela.’

(8a) Na'e fakamaau'i 'e 'Elina 'a Melaia. ‘Elina judged Melaia.’

(8a) Na'e fakamaau'i 'a 'Elina 'e Melaia. ‘Melaia judged ‘Elina.’

(9a) Na'e akonaki'i 'e Siope 'a Soa. ‘Siope disciplined Soa.’

(9b) Na'e akonaki'i 'a Siope 'e Soa. ‘Soa disciplined Siope.’

(10a) Na'e talatalaaki'i 'e Seneti 'a Sifa. ‘Seneti told on Sifa.’

(10b) Na'e talatalaaki'i 'a Seneti 'e Sifa. ‘Sifa told on Seneti.’

(11a) Na'e kumi 'e Make 'a Fane. ‘Make searched for Fane.’

(11b) Na'e kumi 'a Make 'e Fane. ‘Fane searched for Make.’

(12a) Na'e fakasio'i 'e Kevini 'a Senitila. ‘Kevini looked for Senitila.’

(12b) Na'e fakasio'i 'a Kevini 'e Senitila. ‘Senitila looked for Kevini.’

(13a) Na'e fakafoki'i 'e Lisa 'a Mona. ‘Lisa sent off Mona.’

(13b) Na'e fakafoki'i 'a Lisa 'e Mona. ‘Mona sent off Lisa.’

(14a) Na'e lau'i 'e Valu 'a Moana. Valu gossiped about Moana.

(14a) Na'e lau'i 'a Valu 'e Moana. Moana gossiped about Valu.

(15a) Na'e kākāa'i 'e Fifita 'a Tina. ‘Fifita cheated on Tina.’

(15a) Na'e kākāa'i 'a Fifita 'e Tina.‘Tina cheated on Fifita.’

(16a) Na'e fakamālōia'i 'e 'Eniketi 'a Matiu. ‘'Eniketi thanked Matiu.’

(16b) Na'e fakamālōia'i 'a 'Eniketi 'e Matiu. ‘Matiu thanked 'Eniketi.’

(17a) Na'e fakamalohi'i 'e Semisi 'a Kaulave. ‘Semisi forced Kaulave.’

(17b) Na'e fakamalohi'i 'a Semisi 'e Kaulave. ‘Kaulave forced Semisi.’

(18a) Na'e fakahaofi 'e Talita 'a Mina. ‘Talita saved Mina.’

(18b) Na'e fakahaofi 'a Talita 'e Mina. ‘Mina saved Talita.’

(19a) Na'e fafangu 'e Vai 'a Saane. ‘Vai wake up Saane.’

(19b) Na'e fafangu 'a Vai 'e Saane. ‘Saane wake up Vai.’

(20a) Na'e fafanga 'e Selai 'a Mela. ‘Selai fed Mela.’

(20b) Na'e fafanga 'a Selai 'e Mela. ‘Mela fed Selai.’

(21a) Na'e ui 'e Latai 'a Liu. ‘Latai called Liu.’

(21b) Na'e ui 'a Latai 'e Liu. ‘Liu called Latai.’

(22a) Na'e tafulu'i 'e 'Amelia 'a Kisione. ‘'Amelia told off Kisione.’

(22b) Na'e tafulu'i 'a 'Amelia 'e Kisione. ‘Kisione told off 'Amelia.’

(23a) Na'e fakamolemole'i 'e Teisi 'a Kesaia. ‘Teisi forgave Kesaia.’

(23b) Na'e fakamolemole'i 'a Teisi 'e Kesaia. ‘Kesaia forgave Teisi.’

(24a) Na'e puke'i 'e Sekope 'a Tatafu. ‘Sekope got hold of Tatafu.’

(24b) Na'e puke'i 'a Sekope 'e Tatafu. ‘Tatafu got hold of Sekope.’

(25a) Na'e faka'ilo 'e Tevita 'a Sela. ‘Tevita prosecuted Sela.’

(25b) Na'e faka'ilo 'a Tevita 'e Sela. ‘Sela prosecuted Tevita.’

(26a) Na'e holataki'i 'e 'Isileli 'a Loa. ‘'Isileli kidnapped Loa.’

(26b) Na'e holataki'i 'a 'Isileli 'e Loa. ‘Loa kidnapped 'Isileli.’

(27a) Na'e tuli'i 'e Sipa 'a Tea. ‘Sipa chased Tea.’

(27b) Na'e tuli'i 'a Sipa 'e Tea. ‘Tea chased Sipa.’

(28a) Na'e fakahū 'e Lini 'a Satini. ‘Lini jailed Satini.’

(28b) Na'e fakahū 'a Lini 'e Satini. ‘Satini jailed Lini.’

(29a) Na'e fakaafe'i 'e Kalo 'a Meki. ‘Kalo invited Meki.’

(29b) Na'e fakaafe'i 'a Kalo 'e Meki. ‘Meki invited Kalo.’

(30a) Na'e fakalotolahi'i 'e Lotu 'a Lesieli. ‘Lotu encouraged Lesieli.’

(30b) Na'e fakalotolahi'i 'a Lotu 'e Lesieli. ‘Lesieli encouraged Lotu.’

**Appendix 3 – Tongan Stimulus Sentences Used for the Sentence Correctness Decision Task in Experiment 3**

All 32 pairs of correct sentences containing three phrases are listed below. TOP (*ko*) refers to a topicalization maker.

(1a) VSO canonical order

*Na'e kai 'e he fefine 'a e ika*.

V(eat)-PST NP(woman)-ERG NP(fish)-ABS

‘The woman ate the fish.’

(1b) VOS scrambled order

*Na'e kai 'a e ika 'e he fefine*.

V(eat)-PAST NP(fish)-ABS NP(woman)-ERG

(1c) SVO subject topicalization

*Ko e fefine na'a ne kai 'a e ika.*

NP(woman)-TOP V(eat)-PAST NP(fish)-ABS

(1d) OVS object topicalization

*Ko e ika na'e kai 'e he fefine*.

NP(fish)-TOP V(eat)-PAST NP(woman)-ERG

Hereafter, only Tongan sentence pairs and their translations are listed.

(2a) *Na'e huo 'e he tangata'eiki 'a e ngōue.* ‘The old man hoed the garden.’

(2b) *Na'e huo 'a e ngōue 'e he tangata'eiki.*

(2c) *Ko e tangata'eiki na'a ne huo 'a e ngōue.*

(2d) *Ko e ngōue na'e huo 'e he tangata'eiki.*

(3a) *Na'e fakamaau 'e he fine'eiki 'a e fale.* ‘The old woman cleaned the house.

(3b) *Na'e fakamaau 'a e fale 'e he fine'eiki.*

(3c) *Ko e fine'eiki na'a ne fakamaau 'a e fale.*

(3d) *Ko e fale na'e fakamaau 'e he fine'eiki.*

(4a) *Na'e tuli 'e he tamaiki 'a e kulī.* ‘The children (young kids) chased the dog.’

(4b) *Na'e tuli 'a e kulī 'e he tamaiki.*

(4c) *Ko e tamaiki na'a nau tuli 'a e kulī.*

(4d) *Ko e kulī na'e tuli 'e he tamaiki.*

(5a) Na'e taa'i 'e he faiako 'a e ta'ahine. ‘The teacher hit the girl.’

(5b) *Na'e taa'i 'a e ta'ahine 'e he faiako.*

(5c) *Ko e faiako na'a ne taa'i 'a e ta'ahine.*

(5d) *Ko e ta'ahine na'e taa'i 'e he faiako .*

(6a) *Na'e fō 'e he kaungā'api 'a e vala 'uli.* ‘The neighbor washed the dirty clothes.’

(6b) *Na'e fō 'a e vala 'uli 'e he kaungā'api.*

(6c) *Ko e kaungā'api na'a ne fō 'a e vala 'uli.*

(6d) *Ko e vala 'uli na'e fō 'e he kaungā'api.*

(7a) *Na'e tafi 'e he fa'eé 'a e veve.* ‘The mother swept the rubbish.’

(7b) *Na'e tafi 'a e veve 'e he fa'eé.*

(7c) *Ko e fa'eé na'a ne tafi 'a e veve.*

(7d) *Ko e veve na'e tafi 'e he fa'eé.*

(8a) *Na'e haka 'e he fa'ētangata 'a e laise.* ‘The uncle cooked the rice.’

(8b) *Na'e haka 'a e laise 'e he fa'ētangata.*

(8c) *Ko e fa'ētangata na'a ne haka 'a e laise.*

(8d) *Ko e laise na'e haka 'e he fa'ētangata.*

(9a) *Na'e tunu 'e he tamai 'a e puaka.* ‘The father roasted the pig.’

(9b) *Na'e tunu 'a e puaka 'e he tamai.*

(9c) *Ko e tamai na'a ne tunu 'a e puaka.*

(9d) *Ko e puaka na'e tunu 'e he tamai.*

(10a) *Na'e ta'o 'e he ta'ahine 'a e pai.* ‘The girl baked the pie.’

(10b) *Na'e ta'o 'a e pai 'e he ta'ahine.*

(10c) *Ko e ta'ahine na'a ne ta'o 'a e pai.*

(10d) *Ko e pai na'e ta'o 'e he ta'ahine.*

(11a) *Na'e tamate'i 'e he faka'uli 'a e puaka.* ‘The driver killed the pig.’

(11b) *Na'e tamate'i 'a e puaka 'e he faka'uli.*

(11c) *Ko e faka'uli na'a ne tamate'i 'a e puaka.*

(11d) *Ko e puaka na'e tamate'i 'e he faka'uli.*

(12a) *Na'e lau 'e he loea 'a e tohi.*  ‘The lawyer read the book.’

(12b) *Na'e lau 'a e tohi 'e he loea.*

(12c) *Ko e loea na'a ne lau 'a e tohi.*

(12d) *Ko e tohi na'e lau 'e he loea.*

(13a) *Na'e ngaahi 'e he 'enisinia 'a e veeni.* ‘The engineer fixed the van.’

(13b) *Na'e ngaahi 'a e veeni 'e he 'enisinia.*

(13c) *Ko e 'enisinia na'a ne ngaahi 'a e veeni.*

(13d) Ko e veeni na'e ngaahi 'e he 'enisinia.

(14a) Na'e vali 'e he tufunga 'a e puha. ‘The carpenter painted the box.’

(14b) Na'e vali 'a e puha 'e he tufunga.

(14c) Ko e tufunga na'a ne vali 'a e puha.

(14d) Ko e puha na'e vali 'e he tufunga.

(15a) Na'e puhi'i 'e he leka 'a e te'elango ‘The child blew the candle.’

(15b) Na'e puhi'i 'a e te'elango 'e he leka.

(15c) Ko e leka na'a ne puhi'i 'a e te'elango

(15d) Ko e te'elango na'e puhi'i 'e he leka.

(16a) Na'e kosi 'e he tuonga'ane 'a e mūsie. ‘The brother mowed the lawn.’

(16b) Na'e kosi 'a e mūsie 'e he tuonga'ane.

(16c) Ko e tuonga'ane na'a ne kosi 'a e mūsie.

(16d) Ko e mūsie na'e kosi 'e he tuonga'ane.

(17a) Na'e langa 'e he mātu'a 'a e fale. ‘The men built the house.’

(17b) Na'e langa 'a e fale 'e he mātu'a.

(17c) Ko e mātu'a na'a nau langa 'a e fale.

(17d) Ko e fale na'e langa 'e he mātu'a.

(18a) Na'e inu 'e he pēpē 'a e hu'akau. ‘The baby drank the milk.’

(18b) Na'e inu 'a e hu'akau 'e he pēpē.

(18c) Ko e pēpē na'a ne inu 'a e hu'akau.

(18d) Ko e hu'akau na'e inu 'e he pēpē.

(19a) Na'e tui 'e he sōtia 'a e sū. ‘The soldier wore the shoe.’

(19b) Na'e tui 'a e sū 'e he sōtia.

(19c) Ko e sōtia na'a ne tui 'a e sū.

(19d) Ko e sū na'e tui 'e he sōtia.

(20a) Na'e tutu 'e he 'ilamutu 'a e 'ovani. ‘The nephew lighted the oven.’

(20b) Na'e tutu 'a e 'ovani 'e he 'ilamutu.

(20c) Ko e 'ilamutu na'a ne tutu 'a e 'ovani.

(20d) Ko e 'ovani na'e tutu 'e he 'ilamutu.

(21a) Na'e kaiha'asi 'e he tuofefine 'a e peni. ‘The sister stole the pen.’

(21b ) Na'e kaiha'asi 'a e peni 'e he tuofefine.

(21c) Ko e tuofefine na'a ne kaiha'asi 'a e peni.

(21d) Ko e peni na'e kaiha'asi 'e he tuofefine.

(22a) Na'e lālanga 'e he mehikitanga 'a e fala. ‘The aunty weaved the mat.’

(22b) Na'e lālanga 'a e fala 'e he mehikitanga.

(22c) Ko e mehikitanga na'a ne lālanga 'a e fala.

(22d) Ko e fala na'e lālanga 'e he mehikitanga.

(23a) Na'e tuitui 'e he kui fefine 'a e kofu. ‘The grandmother sewed the dress.’

(23b) Na'e tuitui 'a e kofu 'e he kui fefine.

(23c) Ko e kui fefine na'a ne tuitui 'a e kofu.

(23d) Ko e kofu na'e tuitui 'e he kui fefine.

(24a) Na'e fakafonu 'e he tōketa 'a e hina. ‘The doctor filled the bottle.’

(24b) Na'e fakafonu 'a e hina 'e he tōketa.

(24c) Ko e tōketa na'a ne fakafonu 'a e hina.

(24d) Ko e hina na'e fakafonu 'e he tōketa.

(25a) Na'e fakama'a 'e he fānau 'a e loki. ‘The children (offspring) cleaned the room.’

(25b) Na'e fakama'a 'a e loki 'e he fānau.

(25c) Ko e fānau na'a nau fakama'a 'a e loki.

(25d) Ko e loki na'e fakama'a 'e he fānau.

(26a) Na'e to'o 'e he faifekau 'a e pa'anga. ‘The preacher took the money.’

(26b) Na'e to'o 'a e pa'anga 'e he faifekau.

(26c) Ko e faifekau na'a ne to'o 'a e pa'anga.

(26d) Ko e pa'anga na'e to'o 'e he faifekau .

(27a) Na'e fufulu 'e he neesi 'a e peleti. ‘The nurse washed the plates.’

(27b) Na'e fufulu 'a e peleti 'e he neesi.

(27c) Ko e neesi na'a ne fufulu 'a e peleti.

(27d) Ko e peleti na'e fufulu 'e he neesi.

(28a) Na'e kaiha'asi 'e he tamasi'i 'a e pasikala. ‘The boy stole the bicycle.’

(28b) Na'e kaiha'asi 'a e pasikala 'e he tamasi'i.

(28c) Ko e tamasi'i na'a ne kaiha'asi 'a e pasikala.

(28d) Ko e pasikala na'e kaiha'asi 'e he tamasi'i.

(29a) Na'e fūfūu'i 'e he faifakatau 'a e kato. ‘The shopkeeper hid the bag.’

(29b) Na'e fūfūu'i 'a e kato 'e he faifakatau.

(29c) Ko e faifakatau na'a ne fūfūu'i 'a e kato.

(29d) Ko e kato na'e fūfūu'i 'e he faifakatau.

(30a) Na'e fana'i 'e he polisi 'a e kulī. ‘The police shot the dog.’

(30b) Na'e fana'i 'a e kulī 'e he polisi.

(30c) Ko e polisi na'a ne fana'i 'a e kulī.

(30d) Ko e kulī na'e fana'i 'e he polisi.

(31a) Na'e tofi 'e he tamasi'i 'a e keke. ‘The boy cut the cake.’

(31b) Na'e tofi 'a e keke 'e he tamasi'i.

(31c) Ko e tamasi'i na'a ne tofi 'a e keke.

(31d) Ko e keke na'e tofi 'e he tamasi'i.

(32a) Na'e fusi'i 'e he motu'a 'a e maea. ‘The man pulled the rope.’

(32b) Na'e fusi'i 'a e maea 'e he motu'a.

(32c) Ko e motu'a na'a ne fusi'i 'a e maea.

(32d) Ko e maea na'e fusi'i 'e he motu'a.
